# Molecular Surveillance for Vector-Borne Bacteria in Rodents and Tree Shrews of Peninsular Malaysia Oil Palm Plantations

**DOI:** 10.3390/tropicalmed8020074

**Published:** 2023-01-19

**Authors:** Siti Nurul Izzah Mohd-Azami, Shih Keng Loong, Jing Jing Khoo, Nurul Aini Husin, Fang Shiang Lim, Nur Hidayana Mahfodz, Siti Nabilah Ishak, Farah Shafawati Mohd-Taib, Benjamin L. Makepeace, Sazaly AbuBakar

**Affiliations:** 1 Tropical Infectious Diseases Research & Education Centre (TIDREC), Higher Institution Centre of Excellence, Universiti Malaya, Kuala Lumpur 50603, Malaysia; 2 Institute for Advanced Studies (IAS), Universiti Malaya, Kuala Lumpur 50603, Malaysia; 3 Institute of Infection, Veterinary & Ecological Sciences, University of Liverpool, Liverpool L3 5RF, UK; 4 Department of Biological Sciences and Biotechnology, Faculty of Science and Technology, Universiti Kebangsaan Malaysia, Bangi 43600, Malaysia; 5 Kuantan Fisheries Biosecurity Centre, Department of Fisheries Malaysia, Kuantan 25100, Malaysia

**Keywords:** *Bartonella phoceensis*, *Borrelia* spp., infectious disease, *Orientia tsutsugamushi*, *Rattus* spp., *Rickettsia* spp.

## Abstract

Many human clinical cases attributed to vector-borne pathogens are underreported in Malaysia, especially in rural localities where healthcare infrastructures are lacking. Here, 217 small mammals, consisting of rodents and tree shrews, were trapped in oil palm plantations in the Peninsular Malaysia states of Johor and Perak. Species identification was performed using morphological and DNA barcoding analyses, and 203 small mammals were included in the detection of selected vector-borne bacteria. The DNA extracted from the spleens was examined for *Orientia tsutsugamushi*, *Borrelia* spp., *Bartonella* spp. and *Rickettsia* spp. using established PCR assays. The small mammals collected in this study included *Rattus tanezumi* R3 mitotype (*n* = 113), *Rattus argentiventer* (*n* = 24), *Rattus tiomanicus* (*n* = 22), *Rattus exulans* (*n* = 17), *Rattus tanezumi sensu stricto* (*n* = 1) and *Tupaia glis* (*n* = 40). *Orientia tsutsugamushi*, *Borrelia* spp. and *Bartonella phoceensis* were detected in the small mammals with the respective detection rates of 12.3%, 5.9% and 4.9%. *Rickettsia* spp., however, was not detected. This study encountered the presence of both Lyme disease and relapsing fever-related borreliae in small mammals collected from the oil palm plantation study sites. All three microorganisms (*Orientia tsutsugamushi*, *Borrelia* spp. and *Bartonella phoceensis*) were detected in the *R. tanezumi* R3 mitotype, suggesting that the species is a competent host for multiple microorganisms. Further investigations are warranted to elucidate the relationships between the ectoparasites, the small mammals and the respective pathogens.

## 1. Introduction

Peridomestic small mammals such as rodents and scandentids (tree shrews) are ubiquitously found in the tropics [1]. These animals are recognized as the hosts of various zoonotic diseases that pose a serious threat to humanity [2]. In addition to having short life cycles, different species of rodents can be found in sympatry due to their synanthropic behavior. As such, it is anticipated that the increase in contact between humans and rodents may promote pathogen transmission in human- dominated habitats [3]. In fact, vector-borne bacteria such as *Bartonella* spp., *Borrelia* spp., *Orientia tsutsugamushi* and *Rickettsia* spp. have become a health concern in Southeast Asia as they are increasingly implicated in human infections [2,4].

Diseases caused by the *O. tsutsugamushi* (scrub typhus), *Borrelia* spp. (Lyme disease and relapsing fever), *Rickettsia* spp. (typhus and spotted fevers) and *Bartonella* spp. (cat scratch disease and trench fever) commonly manifest as undifferentiated fever with headaches and malaise [5,6,7,8]. Some of these diseases can be complicated and fatal if they are not promptly treated [9,10]. Risk factors for these diseases appear to be associated with the presence of animal hosts (e.g., rodents), location (more prevalent in rural and forested areas), seasonality and climate, as well as certain occupations and human behaviors [11,12,13]. Exposures to pathogens causing these diseases have been detected among the Orang Asli (indigenous people) of Peninsular Malaysia [9,14]. Their settlements near or within forested areas and their lifestyle of forest foraging and hunting for wildlife increase their risk of exposure to zoonotic and vector-borne pathogens [15,16,17].

One of the major factors influencing the risk of vector-borne pathogens is attributed to changes in land use [18]. The increasing demand for palm oil had led to the development of new plantations on previously forested lands. However, this trend was curtailed by the Malaysian government’s pledge to maintain at least 50% forest cover in Peninsular Malaysia [19]. Nevertheless, many plantations are already sharing borders with forests and even residential areas [3]. This allows animals, especially peridomestic animals, to roam freely between the forest, plantations and human habitations, potentially contracting, harboring and transmitting diseases to humans living and working in such areas.

Despite the economic contribution of the palm oil industry to the development of Malaysia, very little is known about the effects of vector-borne diseases carried by peridomestic animals on humans living and working in these plantations. This is compounded by the fact that diseases caused by *O. tsutsugamushi*, *Borrelia* spp., *Bartonella* spp. and *Rickettsia* spp. have undifferentiated symptoms compared to the more commonly reported infections such as dengue fever [10,20]. Hence, there is an urgency to investigate the role of peridomestic animals in the transmission of vector-borne pathogens to better understand the dynamics of disease transmission at the oil palm plantation–human habitation interface. The overall objective of the present study was to determine the presence of selected vector-borne bacteria (*O. tsutsugamushi*, *Borrelia* spp., *Bartonella* spp. and *Rickettsia* spp.) in rodents and tree shrews sampled from two oil palm plantations in Peninsular Malaysia.

## 2. Materials and Methods

### 2.1. To Identify the Rodent and Tree Shrew Species Available at Oil Palm Plantations

Archived specimens from the Tropical Infectious Diseases Research and Education Centre (TIDREC), Universiti Malaya were utilized in this study. They consisted of tissues of small mammals from two sampling sites, viz. at UM Plantations Sdn. Bhd., Johor (an oil palm plantation) and Kampung Tumbuh Hangat, Perak (oil palm plantation bordering paddy fields and human settlements). These samples were collected at different times between December 2018 and December 2019 [21]. Ethical approval was obtained from the Universiti Malaya Institutional Animal Care and Use Committee (G8/01082018/24052018-01/R) and permission to conduct the study at Kampung Tumbuh Hangat, Perak was granted by the Department of Orang Asli Development (JAKOA), Malaysia (JAKOA/PP.30.052Jld13 (32)). Approval for small mammal trapping was also received from the University of Liverpool’s Animal Welfare and Ethics Review Body with reference no. AWC0127.

All small mammals captured were initially identified using morphological analysis [22]. Subsequently, tree shrew and rodent DNA barcoding was performed on DNA extracted from their spleens and other organs. Extracted rodent and tree shrew DNA was subjected to a polymerase chain reaction (PCR) targeting the cytochrome c oxidase I (*COI*) gene to determine the rodent and tree shrew species group [23]. The organs were stored at −80 °C immediately after harvesting and the extracted DNAs were aliquoted into three tubes to avoid multiple freeze-thawing. The primers used are listed in Table 1. Positive controls used were genomic DNAs of *O. tsutsugamushi* strain UT176 received from University of Liverpool, United Kingdom, and *Rickettsia roultii* strain established from a tick cell line in TIDREC. Long oligo DNAs were synthesized for the positive controls of *Borrelia* spp. and *Bartonella* spp. The positive control fragments of the flagellin gene, *flaB* and the citrate synthase gene, *gltA* were obtained from *Borrelia burgdorferi* NC001318.1 (501 bp) and *Bartonella quintana* NC005955 (410 bp), respectively. Nuclease-free water was the negative control used in PCR protocols.

The remaining *COI* amplicons (approximately 20 µℓ each) were purified and subsequently sequenced (Apical Scientific Sdn. Bhd., Seri Kembangan, Malaysia). The DNA sequences obtained were trimmed and compared to those available in GenBank using the Basic Local Alignment Search Tool (BLAST). Each identified species was deposited into the GenBank accordingly.

### 2.2. To Detect the Presence of Vector-Borne Bacteria in the Rodents and Tree Shrews Captured in Oil Palm Plantations

About 10 mg of each spleen tissue of the rodents and tree shrews was subjected to DNA extraction following the NucleoSpin^®^ Tissue Extraction Kit (Macherey-Nagel, Düren, Germany) protocol. The extracted genomic DNA was utilized to amplify genes specific for *O. tsutsugamushi*, *Borrelia* spp., *Bartonella* spp. and *Rickettsia* spp. The types of surface antigen 47 kDa gene *TSA47* specific to *O. tsutsugamushi* [24] and *flaB* specific to the *Borrelia* spp. [25] were amplified according to previously published protocols. The detection of *Bartonella* spp. and *Rickettsia* spp. followed two different PCR protocols that target *gltA* [26,27,28]. Primers used in the present study are listed in Table 1.

The PCR-positive DNA samples for *O. tsutsugamushi* and *Borrelia* spp. were further subjected to multi-locus sequence typing (MLST) following the protocols for *Borrelia* spp. [29] and *O. tsutsugamushi* [30]. These protocols are available at their respective PubMLST databases (https://pubmlst.org/organisms/borrelia-spp (accessed on 13 October 2021) and https://pubmlst.org/organisms/orientia-tsutsugamushi (accessed on 13 October 2021). All obtained amplicons were purified and subsequently sequenced in both directions by a third party (Apical Scientific Sdn. Bhd., Malaysia). The DNA sequences obtained were trimmed and compared to those available in GenBank and PubMLST.

**Table 1:** Primers used for DNA barcoding and pathogen detection.

Organism	Target	Primer	Oligonucleotide Sequence (5′-3′)	Amplicon Size (bp)	Reference
Rodents	*COI*	BatL5310 ^a,c^	ACTTCTGGGTGTCCAAAGAATCA	726	[23]
		R6036R ^b,c^	CCTACTCRGCCATTTTACCTATG		
*Orientia tsutsugamushi*	*TSA47*	Ot-145F ^a^	ACAGGCCAAGATATTGGAAG	871	[24]
		Ot-1780R ^b^	AATCGCCTTTAAACTAGATTTACTTATTA		
		Ot-263F ^a,c^	GTGCTAAGAAARGATGATACTTC	821	
		Ot-1133R ^b,c^	ACATTTAACATACCACGACGAAT		
*Bartonella* spp.	*gltA*	BhCS.781p ^a,c^	GGGGACCAGCTCATGGTGG	379	[28]
		BhCS.1137n ^b,c^	AATGCAAAAAGAACAGTAAACA		
*Borrelia* spp.	*flaB*	BflaPAD ^a^	GATCARGCWCAAYATAACCAWATGCA	800	[25]
		BflaPDU ^b^	AGATTCAAGTCTGTTTTGGAAAGC		
		BflaPBU ^a,c^	GCTGAAGAGCTTGGAATGCAACC	345	
		BflaPCR ^b,c^	TGATCAGTTATCATTCTAATAGCA		
*Rickettsia* spp.	*gltA*	CS1d ^a,c^	ATGACTAATGGCAATAATAA	889	[26]
		CS890r ^b,c^	GCTTTIAGCTACATATTTAGG		
		CS-239 ^a,c^	GCTCTTCTCATCCTATGGCTATTAT	830	[27]
		CS-1069 ^b,c^	CAGGGTCTTCGTGCATTTCTT		

a—Forward primer, b—reverse primer, c—sequencing primer.

### 2.3. To Determine the Genetic Relatedness of the Detected Bacteria to Well-Characterized Counterparts

Following bacteria identification using the BLAST tool, the primer-trimmed sequences of the respective targeted genes were aligned using CLUSTALW, as implemented in MEGAX [31]. All positions containing gaps and missing data were eliminated (complete deletion option). Phylogenetic relationships of the pathogens detected in this study were presented in phylogenetic trees using the Bayesian Markov Chain Monte Carlo (MCMC) approach, as implemented in BEAST 1.10.4 [32]. The Hasegawa–Kishono–Yano (HKY) model with the Gamma site (HKY + G) was selected for all the targeted genes using the Bayesian Information Criterion (BIC) as implemented in MEGA11 [33]. The analysis was performed under a strict molecular clock model with an MCMC chain length of 5 million samplings every 1000 generations. The resulting MCMC trace file was analyzed and visualized using Tracer Version 1.7.1 (Institute of Evolutionary Biology, University of Edinburgh, UK) [34]. The maximum clade credibility (MCC) tree was produced using TreeAnnotator 1.10.4 (Institute of Evolutionary Biology, University of Edinburgh, UK) and visualized using the Interactive Tree of Life (iTOL) (https://itol.embl.de/itol.cgi (accessed on 29 December 2022). A pairwise comparison analysis, as implemented in MEGA11, was conducted for the *O. tsutsugamushi* sequences obtained after the phylogenetic analyses were completed.

## 3. Results

### 3.1. Distribution of Small Mammal Species

The morphological identification conducted on the tree shrews (*n* = 40) resulted in the identification of a single species, *Tupaia glis*. The DNA barcoding revealed the identification of five separate rodent species: *Rattus tanezumi* R3 mitotype (*n* = 113), *Rattus argentiventer* (*n* = 24), *Rattus tiomanicus* (*n* = 22), *Rattus exulans* (*n* = 17) and *Rattus tanezumi sensu stricto* (*s.s.*) (*n* = 1) (Table 2).

The *R. tanezumi* R3 mitotype (*n* = 113, 52.1%) predominated in both sites followed by *T. glis* (*n* = 40, 18.4%), *R. argentiventer* (*n* = 24, 11.1%), *R. tiomanicus* (*n* = 22, 10.1%), *R. exulans* (*n* = 17, 7.8%) and *R. tanezumi s.s.* (*n* = 1, 0.5%). Both sites had a similar number of small mammals trapped. In Johor, *T. glis* (*n* = 33) outnumbered *R. tiomanicus* (*n* = 13) and *R. exulans* (*n* = 3), while *R. tanezumi s.s* and *R. argentiventer* were not found. In Perak, *R. tanezumi s.s.* was solely found in the paddy field, while *R. tiomanicus* and *T. glis* were absent there. Additionally, *R. argentiventer* was absent in the residential areas. Out of the 217 trapped animals, 105 of them were females and 112 of them were males. The majority of the captured animals were mature adults (*n* = 148, 68.2%) and subadults (*n* = 41, 18.9%), followed by juveniles (*n* = 25, 11.5%); the age of the remaining 3 individuals could not be ascertained. 

The *R. tanezumi* R3 mitotype was found in all habitats, but predominantly in the oil palm plantations. All the successful *COI* sequences of the rodents were deposited into the Barcode of Life Data Systems (BOLD) (http://boldsystems.org (accessed on 14 January 2022) under the project code UMNPA as described in a previous study [21].

### 3.2. PCR Detection of Bacteria in Small Mammals

The DNA extracted from the spleens of 203 small mammals (rodents, *n* = 163 and tree shrews, *n* = 40) was examined using the pathogen-specific PCR for the presence of *O. tsutsugamushi*, *Borrelia* spp., *Bartonella* spp. and *Rickettsia* spp. (Table 3). Rodent splenic tissues insufficient for DNA extraction were excluded from the study (*n* = 14). The PCR assays targeted the *TSA47* gene for *O. tsutsugamushi*, the *gltA* gene for *Bartonella* spp. and *Rickettsia* spp. and the *flaB* gene for *Borrelia* spp. Overall, 12.3% (25/203) of the small mammals were positive for the presence of *O. tsutsugamushi* followed by *Borrelia* spp. at 5.9% (12/203) and *Bartonella phoceensis* at 4.9% (10/203). *Rickettsia* spp., however, was not detected in any specimen. 

The bacteria detection rate was higher in Perak (15.8%) compared to Johor (7.4%). *Orientia tsutsugamushi* was detected in all small mammal species except for *R. tanezumi s.s*. *Borrelia* spp. was detected in four species but not for *R. tanezumi s.s.* and *R. argentiventer*, while *B. phoceensis* was detected only in the *R. tanezumi* R3 mitotype and *R. argentiventer*. *Orientia tsutsugamushi* was detected most frequently in the *R. tanezumi* R3 mitotype at both study sites (Perak, *n* = 11; Johor, *n* = 7) (Table 3). *Bartonella phoceensis* and *Borrelia* spp. were the second most detected bacteria in Perak (*n* = 9) and Johor (*n* = 4). Furthermore, there were four individuals co-infected with *B. phoceensis* and *O. tsutsugamushi,* with three from Perak and one from Johor. 

### 3.3. Sequence Analyses of the Detected Bacteria

#### 3.3.1. *Orientia tsutsugamushi*

**Figure 1:**
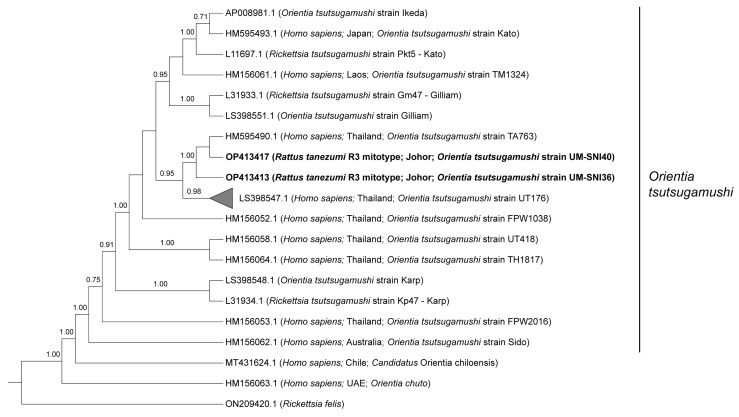
Bayesian inference phylogenetic tree of *O. tsutsugamushi* based on the partial sequences (825 bp) of the *TSA47* gene. Posterior probability (PP) is shown on the branches. Only PP > 0.7 are shown. Newly generated sequences are in bold text, with their accession numbers followed by the animal host species, location and strain name in parentheses. The reference sequences are labelled with their accession numbers followed by the host, location and *O. tsutsugamushi* strain in parentheses (some only contain partial information). The collapsed branch, consisting of the 23 new sequences from this study, clustered together with strain UT176 (Accession no. LS398547.1) at 0.98 PP.

Phylogenetic analyses of the 825 bp sequences from the *O. tsutsugamushi TSA47*-positive specimens grouped all of them together with two strains reported in Thailand (UT176 and TA763) at 0.95 posterior probability (PP) (Figure 1). Sequences from the current study (UM-SNI36 and UM-SNI40) clustered with the *O. tsutsugamushi* strain TA763 (1.00 PP). The remaining 23 specimens that were clustered with the *O. tsutsugamushi* strain UT176 (0.98 PP) had pairwise distances ranging from 0 to 1.61% between them. Out of seven genes from the *O. tsutsugamushi* MLST scheme, we only managed to amplify the succinyl-CoA synthetase (*sucD*) and pyruvate phosphate dikinase precursor (*ppdK*) genes from one *R. tanezumi* R3 mitotype host. These sequences, however, could not be deposited into the PubMLST database for *O. tsutsugamushi* as there were several polymorphic double peaks in the respective chromatograms (Supplementary Figures S1 and S2). Subsequent BLASTn analyses based on the most dominant chromatogram signals revealed that the amplified *ppdK* and *sucD* sequences were identical to *O. tsutsugamushi* isolate Karp (Accession no. LS398548.1) at 100% and the *O. tsutsugamushi* strain Wuj/2014 (Accession no. CP044031.1) at 98.9% identities, respectively.

#### 3.3.2. *Borrelia* spp.

**Figure 2:**
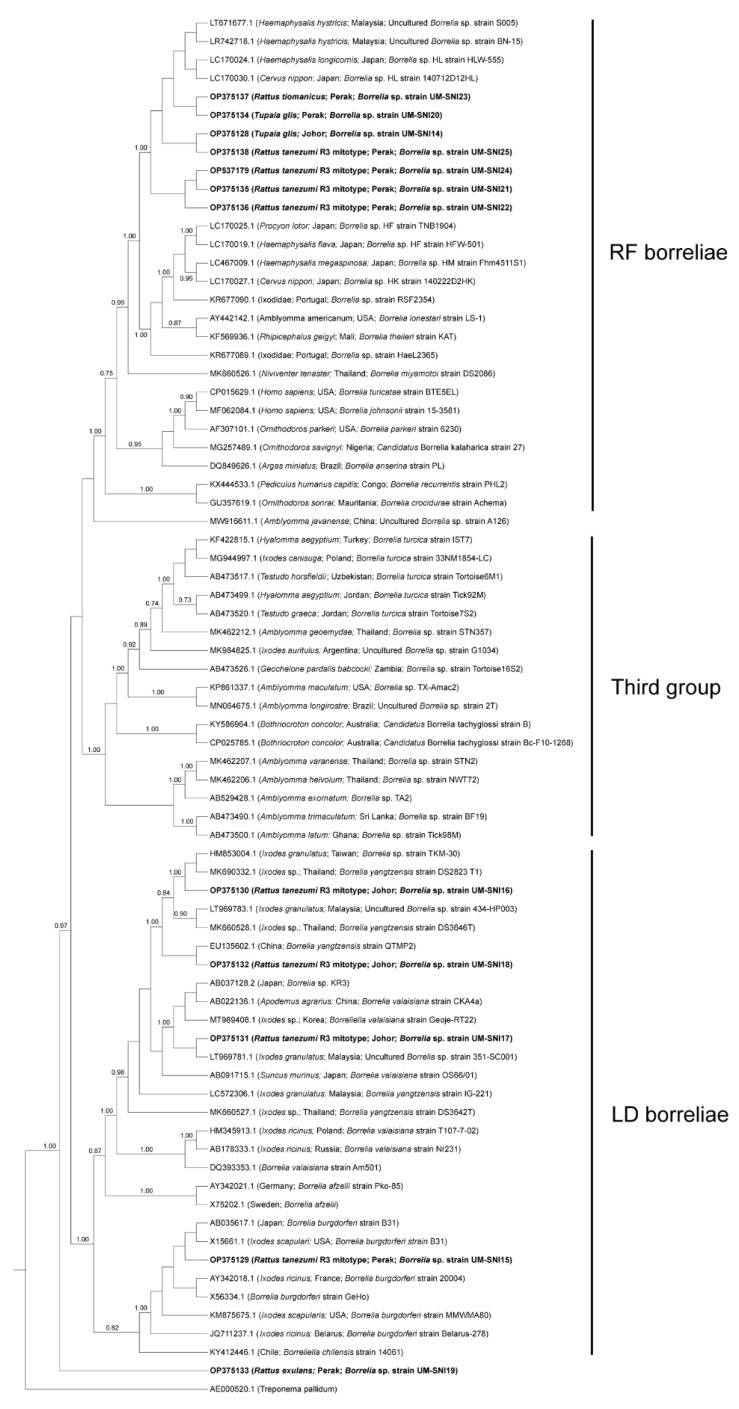
Bayesian inference phylogenetic tree of *Borrelia* spp. based on the partial sequences (270–300 bp) of the *flaB* gene. Posterior probability (PP) is shown on the branches. Only PP > 0.7 are shown. Newly generated sequences are in bold text, with their accession numbers followed by the animal host species, location and strain name in parentheses. The reference sequences are labelled with their accession numbers followed by the host, location and *Borrelia* spp. strain in parentheses (some only contain partial information). LD = Lyme disease-related, RF = relapsing fever-related.

The borrelial *flaB* sequences generated from this study were segregated into two clusters, one with members of the Lyme disease-related (LD) borreliae and the other with members of the relapsing fever-related (RF) borreliae (Figure 2), consistent with previous reports [35,36,37]. A third cluster whose members did not belong to the former two groups was also included in the analysis, but none of our specimens clustered with this group. Both LD and RF borreliae were detected in specimens collected from both study sites (Figure 2). LD borreliae were only detected in the *R. tanezumi* R3 mitotype (*n* = 4) captured in Perak and Johor. In contrast, the RF borreliae were detected in several species such as the *R. tanezumi* R3 mitotype (*n* = 4), *R. tiomanicus* (*n* = 1) and *T. glis* (*n* = 1) captured in Perak and *T. glis* captured in Johor (*n* = 1) (Table 3).

A closer observation of the RF borreliae obtained from this study suggested that they form a sister clade independent of the other RF borreliae strains. This clade includes the unculturable *Borrelia* spp. detected in Malaysia (Accession nos. LT671677.1 and LR742718.1) and Japan (Accession nos. LC170024.1 and LC170030.1) (Figure 2). Conversely, the LD borreliae topology showed that most of our specimens clustered with *Borrelia yangtzensis* and *Borrelia valaisiana* genospecies group members reported from other Asian countries.

One specimen (UM-SNI15) was clustered with various strains of *B. burgdorferi*, including the *B. burgdorferi sensu stricto* (*s.s.*) strains B31 and 20004, isolated from *Ixodes* spp. ticks in the USA and France, respectively [38,39]. A novel *clpA* allele (Allele 310) was obtained following the *Borrelia* spp. MLST scheme but the other genes failed to be amplified. The successfully amplified specimen for MLST was collected from one *R. tanezumi* R3 mitotype from Johor (UM-SNI18). It was found to be genetically related to *B. yangtzensis* (Accession no. LC572085.1) at 98.63% identity.

Notably, specimen UM-SNI19 was separately distinct from all other groups, forming a sister clade next to the other LD borreliae members with less than 0.7 PP. BLASTn analysis showed that UM-SNI19 has less than 90% identity to both LD and RF borreliae.

#### 3.3.3. *Bartonella* spp.

**Figure 3:**
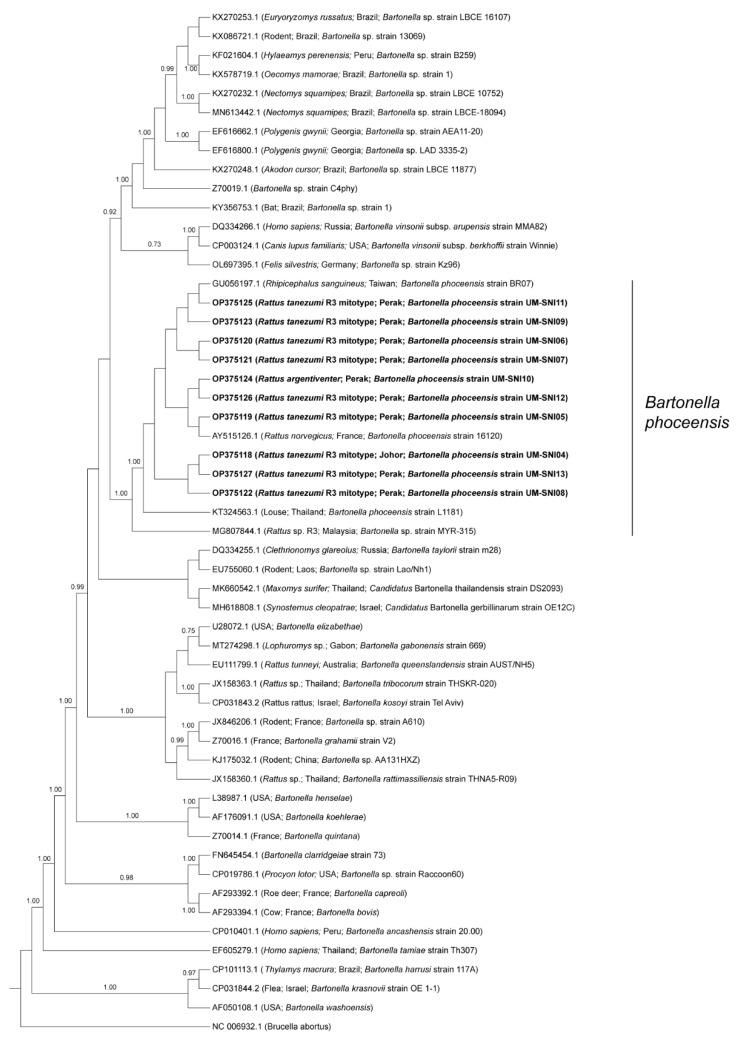
Bayesian inference phylogenetic tree of *B. phoceensis* based on the partial sequences (338 bp) of the *gltA* gene. Posterior probability (PP) is shown on the branches. Only PP > 0.7 are shown. Newly generated sequences are in bold text, with their accession numbers followed by the animal host species, location and strain name in parentheses. The reference sequences are labelled with their accession numbers followed by the host, location and *Bartonella* spp. strain in parentheses (some only contain partial information).

BLAST analyses of the amplified sequences specific to the *gltA* of *Bartonella* spp. revealed that all the specimens were positive for *B. phoceensis* with 99–100% similarities. The phylogenetic tree displayed the clustering of the specimens from this study into one clade together with *B. phoceensis* representatives from different geographical locations and separate from the other *Bartonella* spp. (Figure 3).

## 4. Discussion

In the present study, small mammals (rodents and tree shrews) trapped in two locations, Johor and Perak, were studied for the presence of selected vector-borne bacteria (*O. tsutsugamushi*, *Borrelia* spp., *Bartonella* spp. and *Rickettsia* spp.) by the PCR amplification of pathogen-specific genes. The dominant ecotype in both study sites was the oil palm plantation. Five rodent (*R. tanezumi* R3 mitotype, *R. exulans, R. tiomanicus, R. argentiventer* and *R. tanezumi* s.s.) species and one tree shrew (*T. glis*) species were found in the study sites. *Orientia tsutsugamushi*, LD and RF borreliae and *B. phoceensis* were detected in most of the small mammals except for *R. tanezumi s.s.* (*n* = 1), while *Rickettsia* spp. was not detected at all. 

*Orientia tsutsugamushi* has been detected in small mammal species across Southeast Asia (reviewed in [40]), and detection was usually based on bacteria isolation or serology [41,42,43,44,45,46,47,48]. However, more recent efforts focused on PCR assays targeting the *TSA47* gene for *O. tsutsugamushi* detection as they are more sensitive and the products can be sequenced to provide genetic information [49,50,51,52]. In the present study, *O. tsutsugamushi* was detected in 12.3% of the small mammals. This detection rate was higher than in a previous study that detected it in only about 1% of small mammals captured from eight different states in Malaysia [50]. Another study employing the PCR detection of *O. tsutsugamushi* in rodents captured near the Selangau Health Center, Sarawak, Malaysia, did not yield any positive results [53]. The highest prevalence to our knowledge was the 20% *O. tsutsugamushi*–positive detection in the liver and spleen of rodents sampled in Si Racha, Chonburi province, Thailand [47]. Apart from that, most studies resulted in very low *O. tsutsugamushi* infection rates ranging from 0.7 to 2.3%, as compared to our study. Those studies also employed the PCR method, but they were detecting the pathogen in different tissues (e.g., kidneys) as opposed to spleen, which could explain the differences in detection rates [46,54,55,56]. Multiple vector-borne pathogens have been detected in the spleen as opposed to other tissues, making the spleen the targeted tissue in the present study [57].

In contrast to our phylogenetic tree in Figure 1, the UT176 strain has been reported as the Karp sub-genotype with TA763 as a separate genotype based on the *TSA56* genotyping [49]. In Thailand, eight clades have been known to circulate, viz. Karp, Kato, Gilliam, TA678, TA686, TA716, TA763 and TH1817 since the 1960s [51,58,59]. In addition, an epidemiology study revealed that at least five genotypes were circulating in Cambodia and three in Vietnam [60]. During inspection of the *TSA47* sequencing chromatograms, we noticed double peaks (i.e., two different bases) at some nucleotide positions. However, those sequences were excluded from the analyses in this study.

A majority of *O. tsutsugamushi* surveys in small mammals were conducted in Thailand. These studies reported the positive detection of *O. tsutsugamushi* in the *Rattus rattus* complex, *Bandicota indica*, *T. glis*, *R. tanezumi*, *Rattus andamanensis*, *R. exulans*, *Mus cookii*, *Rattus nitidus*, *Bandicota savilei*, *Berylmys berdmorei*, *Berylmys bowersi*, *Leopoldamys edwardsi* and *Rattus* sp. phylogenetic clade 3, as well as chiggers associated with small mammals [42,46,47,55,56,61]. In Vietnam, *O. tsutsugamushi* was detected in *Rattus flavipectus* [61] and *Rattus norvegicus* [62] suggesting that rodents and tree shrews are potential competent reservoirs for *O. tsutsugamushi*.

A recent study reported the detection of *O. tsutsugamushi* in chiggers parasitizing *R. rattus* and *Tupaia* sp. in Malaysia, albeit from a different state, Kelantan [63]. The animal hosts were trapped in areas near the house of a scrub typhus patient, surrounded by mixed ecologies such as shrubs, coconut, fruit and sugar cane orchards. The study also reported that two of sixteen pools of *Leptotrombidium deliense* mites (12.5%) tested positive for *O. tsutsugamushi* [63]. Chaisiri et al. [54] reported that *O. tsutsugamushi*-infected rodents in Thailand were also obtained from similar ecotypes such as forested and reforestation areas, fallows, cassava plantations and rice fields. Although the main ecotype covered in our study was the oil palm plantation, there were rice fields and residential areas near the Perak study site. Our previous ecological analysis of *O. tsutsugamushi* infection in the same rodents analyzed in the current study concluded that neither habitat nor season was significantly associated with infection, although infection prevalence was highest in oil palm plantations compared with peripheral habitats [64]. This lack of statistically significant ecological effects may be due to the long duration of *O. tsutsugamushi* infection in small mammals or high reinfection rates coupled with their movement between adjacent habitats [40]. In Johor, the *O. tsutsugamushi* infection of small mammals was positively associated with a Malaysian endemic vector, *Leptotrombidium arenicola*, although no significant relationship between any chigger species and *O. tsutsugamushi* infection in Perak was apparent [64].

*Orientia tsutsugamushi* was detected in all small mammal species collected from this study except for *R. tanezumi s.s.*, and this can be explained as *R. tanezumi s.s.* has been shown to be an incidental rodent species in oil palm plantations in Malaysia [65]. The two synanthropic species, *R. exulans* and the *R. tanezumi* R3 mitotype, live in close association with humans [66,67,68]. From our findings, both species were found to carry *O. tsutsugamushi*. The current study also detected *O. tsutsugamushi TSA47* sequences similar to the *O. tsutsugamushi* isolated from scrub typhus patients [49,69,70]. This was congruent to studies reporting the infection of *O. tsutsugamushi* in febrile patients, healthy villagers and rubber estate workers from rural areas [71,72,73]. Moreover, polymorphic double peaks were observed in the chromatograms for *ppdK* and *sucD* (Supplementary Figures S1 and S2), suggesting the presence of more than one *O. tsutsugamushi* strain in the specimen. A similar observation was noted in scrub typhus patients; researchers found that some patients could be simultaneously infected with multiple *O. tsutsugamushi* strains [30]. Altogether, these studies imply the potential risk of scrub typhus being contracted by inhabitants or workers in the agriculture sector such as oil palm plantations, where there could be exposure to chiggers and small mammal hosts.

The RF borreliae from the present study were found to be closely related to the *Borrelia* sp. detected from Japanese sika deer (*Cervus nippon*) and its associated tick ectoparasite, *Haemaphysalis longicornis*) [74,75,76], a tick species not reported in Malaysia. Several strains from the present study also clustered with RF borreliae that were previously detected in *Haemaphysalis hystricis* collected from a wild boar [35] and a dog [77]. These findings suggest that both *H. longicornis* and *H. hystricis* might harbor closely related borrelial strains. Our study presented evidence of the detection of RF borreliae strains in *Rattus* spp. rodents and *T. glis*. The majority of RF borreliae strains in this study were detected in Perak. In Thailand, a previous study reported the detection of RF borreliae in rodents (*Rattus* spp., *B. indica*, *Niviventer* spp., *Leopoldamys sabanus*, *Crocidura fuliginosa*, *Mus caroli* and *M. cookii*) and ticks (*Haemaphysalis bandicota*, *Rhipicephalus sanguineus*, *Ixodes granulatus* and *Dermacentor* spp.). They were closely related to *Borrelia theileri*, *Borrelia lonestari* and *Borrelia miyamotoi* [56,78]. *Borrelia crocidurae*, the causative agent of tick-borne relapsing fever in West Africa, was commonly detected in small mammals, suggesting their importance in the disease epidemiology [79,80]. Small mammals were also reported as potential reservoirs for *B. miyamotoi*, another RF borreliae, in different geographical regions including Malaysia [25,81,82]. The findings from our study add to the evidence of the role of small mammals, especially the *Rattus* spp. rodents and *T. glis*, in the ecology and maintenance of the identified RF borreliae in the studied areas. *B. miyamotoi* was previously assumed to be non-pathogenic until the first human infection was reported in Russia [83]. Although the currently identified RF borreliae strains have yet to be associated with human infections, increased surveillance is important as small mammal infestation is widespread in oil palm plantations, which may lead to eventual pathogen transmission to humans residing or working within the plantations.

To our knowledge, the data presented here are the first findings of borrelial sequences related to *B. burgdorferi s.s.* from rodents in Southeast Asia. Sequences related to *B. burgdorferi s.s.* were previously detected in the blood of a dog from Thailand [84]. Similarly, a sequence closely related to *B. burgdorferi s.s.* was detected in one rodent from Perak in the present study (UM-SNI15). Although the pathogen was detected in only one specimen, more surveillance effort is required to establish the presence of *B. burgdorferi s.s.* in Malaysia, and to identify the tick vector. While we only managed to sequence the *clpA* allele following the *Borrelia* spp. MLST scheme, a novel *clpA* allele 310 was assigned to it, indicating a novel *Borrelia* strain. The *clpA* allele 310 was most closely related to *clpA* allele 81 that belongs to *B. yangtzensis* ST360 (Supplementary Figures S3 and S4); these strains were isolated from the *M. caroli* rodent and *I. granulatus* tick in Japan [85]. *B. yangtzensis* and the related strains are commonly associated with rodents and tick ectoparasites. From this study, all strains closely related to *B. yangzentsis* were detected in Johor. In Malaysia, *B. yangtzensis*-related strains were first reported in *I. granulatus* ticks collected from *Sundamys muelleri* in a recreational forest [36,86]). Furthermore, a previous northern Thailand study reported the detection of *B. yangtzensis* in rodents and the associated *Ixodes* tick and chigger ectoparasites [56]. *B. yangtzensis* was also detected in rodents and their tick ectoparasites in China and Japan [87]. This indicates that *B. yangtzensis*-related strains are widespread in East and Southeast Asia. Additionally, the findings from our study included the *R. tanezumi* R3 mitotype as another potential host for the pathogen. Since *B. yangtzensis* is pathogenic to humans [88], our findings suggest that *B. yangtzensis* could put residents of oil palm plantations at risk of infection.

We were unable to ascertain the phylogenetic placement of one of the borrelial sequences (UM-SNI19) in this study. BLASTn analyses suggest that UM-SNI19 may be more closely related to the RF borreliae as the highest query cover and identity scores matched with members of the RF borreliae strains, even though a portion of the sequences also exhibited a high percentage of identity to a single member of the LD borreliae, *Borrelia afzelii*. Moreover, this strain contains a unique gap in the *flaB* sequences compared to other strains in the multiple sequence alignment provided in Supplementary Figure S5. These findings suggest that the UM-SNI19 *Borrelia* sp. may be a distinctive genotype based on the *flaB* sequences. However, investigation into more genes and more specimens will be necessary to confirm this observation.

The prevalence of *B. phoceensis* amongst the small mammals in this study (4.9%) was relatively low compared to a study by [89] in Sarawak, Malaysia. They found that approximately 25% of the total examined rodents were *B. phoceensis*-positive. Their study also found that the prevalence of *B. phoceensis* was lower in rural areas, concurring with our observations. The authors suggest that *Bartonella* spp. are less prevalent in rural areas due to the larger foraging habitats, discouraging contact between rodents, thus reducing *Bartonella* spp. transmission [89]. The detection of *B. phoceensis* among small mammals in Malaysia has previously been reported by Low et al. [90] and Asyikha et al. [91]. *Bartonella phoceensis* was found in small mammals captured from urban and rural areas, suggesting that the pathogen is prevalent in small mammal hosts from various habitats [89,90,91]. A separate study reported the detection of *Bartonella* spp. in the blood of several rodent species, including *Rattus* spp. [13], similar to the present study that detected *B. phoceensis* in the *R. argentiventer* and *R. tanezumi* R3 mitotype. Even though the pathogenicity of *B. phoceensis* to humans has not been established, the bacterium has been detected in mites, lice and ticks associated with rodents [92], suggesting the risk of transmission to humans. Additionally, four *R. tanezumi* R3 mitotypes from the Johor and Perak study sites were found to be co-infected with *O. tsutsugamushi* and *B. phoceensis*, compounding the transmission risk. Nevertheless, this finding is not surprising as rodents are frequently coinfected with multiple pathogens [45].

## 5. Conclusions

We report here the presence of *O. tsutsugamushi*, LD and RF borreliae and *B. phoceensis* amongst small mammals commonly found in oil palm plantations in Johor and Perak, Malaysia. Our findings include a potentially novel *Borrelia* genotype, and the first report of a *Borrelia* sp. closely related to *B. burgdorferi s.s.* in a rodent in this country. *Orientia tsutsugamushi* and *B. phoceensis* were detected together in four *R. tanezumi* R3 mitotype hosts, indicating the simultaneous presence of different pathogens in the rodents. The findings from this study suggest that *O. tsutsugamushi*, *Borrelia* spp. and *B. phoceensis* are prevalent among the small mammal populations. The fact that these animals are found in abundance in the oil palm plantation and can harbor multiple pathogens increases the risk of potential transmission to other animals, including humans, in the vicinity.

## Figures and Tables

**Table 2:** The identification of small mammals trapped in Perak and Johor.

No.	Species	Trapping Site				Total Number of Individuals
		Perak (*n*)			Johor (*n*)	
		Residential Areas	Paddy Field	Oil Palm Plantation	Oil Palm Plantation	
1.	*Rattus tanezumi* R3 mitotype	14	2	45	52	113
2.	*Rattus tiomanicus*	2	0	7	13	22
3.	*Rattus exulans*	3	2	9	3	17
4.	*Rattus tanezumi sensu stricto*	0	1	0	0	1
5.	*Rattus argentiventer*	0	21	3	0	24
6.	*Tupaia glis*	3	0	4	33	40
Total number of individuals		116			101	217

**Table 3:** Vector-borne bacteria detected from the spleens of rodents and tree shrews.

Location	Host Species	Detected Vector-Borne Bacteria	Number of Positive Individuals (*n*)
Perak	*Rattus tanezumi* R3 mitotype	*Bartonella phoceensis*	8
		*Orientia tsutsugamushi*	11
		*Borrelia sp.* (LD)	1
		*Borrelia sp.* (RF)	4
	*Rattus exulans*	*Borrelia sp.* (undetermined)	1
		*Orientia tsutsugamushi*	2
	*Rattus argentiventer*	*Bartonella phoceensis*	1
		*Orientia tsutsugamushi*	2
	*Rattus tiomanicus*	*Borrelia sp.* (RF)	1
	*Tupaia glis*	*Borrelia sp.* (RF)	1
Johor	*Rattus tanezumi* R3 mitotype	*Bartonella phoceensis*	1
		*Orientia tsutsugamushi*	7
		*Borrelia sp.* (LD)	3
	*Rattus tiomanicus*	*Orientia tsutsugamushi*	1
	*Tupaia glis*	*Orientia tsutsugamushi*	2
		*Borrelia sp.* (RF)	1

LD = Lyme disease-related; RF = relapsing fever-related.

## Data Availability

The rodent (*Rattus* sp.) *COI* sequences generated in this study were deposited in the Barcode of Life Data System (BOLD) under the process IDs, UMNPA004-20 - UMNPA056-20, and UMNPA058-20 - UMNPA068-20 for rodents captured from Johor, and UMNPA069-20, UMNPA071-20 - UMNPA076-20, UMNPA078-20 - UMNPA080-20, UMNPA082-20 - UMNPA083-20, UMNPA085-20, UMNPA087-20 - UMNPA091-20, UMNPA093-20 - UMNPA102-20, UMNPA161-20 - UMNPA194-20, UMNPA196-20 - UMNPA216-20, and UMNPA218-20 - UMNPA223-20 for rodents captured from Perak. The *Orientia*-specific *TSA47* gene amplicon sequences generated in this study were deposited in NCBI Genbank with accession numbers OP413403 - OP413427. The *Borrelia*-specific *flaB* gene amplicon sequences generated in this study were deposited in NCBI Genbank under the accession numbers OP375128 - OP375138 and OP537179. The *Bartonella*-specific *gltA* gene amplicon sequences generated in this study were deposited in NCBI Genbank with accession numbers OP375118 - OP375127. The *clpA* allele sequences for the *Borrelia* strain from this study can be accessed at the PubMLST database (https://pubmlst.org/organisms/borrelia-spp, accessed on 16 January 2023).

## Supplementary Materials

The following supporting information can be downloaded at https://www.mdpi.com/article/10.3390/tropicalmed8020074/s1. Figure S1: Positions with heterozygous double peaks in the *sucD* allele of *Orientia tsutsugamushi*, Figure S2: Positions with heterozygous double peaks in the *ppdK* allele of *Orientia tsutsugamushi*, Figure S3: Similarities of *clpA* allele 310 (*Borrelia yangtzensis*) with closely related *clpA* alleles in the PubMLST database, Figure S4: *Borrelia yangtzensis* ST360 strains deposited in the PubMLST database, Figure S5: The unique gap in the *flaB* sequences of *Borrelia* sp. strain UM-SNI19 (red box), compared to other borreliae strains, Table S1: The morphological information of small mammals trapped in Johor and Perak, Malaysia.

## References

[1] Muul I., Lim B.L., Walker J.S. (1977). Scrub typhus infection in rats in four habitats in Peninsular Malaysia. Trans. R. Soc. Trop. Med. Hyg..

[2] Meerburg B.G., Singleton G.R., Kijlstra A. (2009). Rodent-borne diseases and their risks for public health. Crit. Rev. Microbiol..

[3] Morand S., Blasdell K., Bordes F., Buchy P., Carcy B., Chaisiri K., Chaval Y., Claude J., Cosson J.F., Desquesnes M. (2019). Changing landscapes of Southeast Asia and rodent-borne diseases: Decreased diversity but increased transmission risks. Ecol. Appl..

[4] Shah H.A., Huxley P., Elmes J., Murray K.A. (2019). Agricultural land-uses consistently exacerbate infectious disease risks in Southeast Asia. Nat. Commun..

[5] Karski J., Okoński M., Pietrzyk D., Karska K., Zaluski M. (2018). Cat scratch disease in a 1.5-year-old girl-case report. Ann. Agric. Environ. Med..

[6] Luce-Fedrow A., Lehman M.L., Kelly D.J., Mullins K., Maina A.N., Stewart R.L., Ge H., John H.S., Jiang J., Richards A.L. (2018). A review of scrub typhus (*Orientia tsutsugamushi* and related organisms): Then, now, and tomorrow. J. Trop. Med. Infect. Dis..

[7] Pun S.B., Agrawal S., Jha S., Bhandari L.N., Chalise B.S., Mishra A., Shah R. (2018). First report of Lyme disease in Nepal. JMM Case Rep..

[8] Warrell D.A. (2019). Louse-borne relapsing fever (*Borrelia recurrentis* infection). Epidemiol. Infect..

[9] Khor C.S., Hassan H., Mohdrahim N., Chandren J., Nore S.S., Johari J., Loong S.K., Abd-Jamil J., Khoo J.J., Lee H. (2019). Seroprevalence of *Borrelia burgdorferi* among the indigenous people (Orang Asli) of Peninsular Malaysia. J. Infect. Dev. Ctries..

[10] Loong S.K., Abd-Majid M.A., Teoh B.T., Cheh M.J., Khor C.S., Chao C.C., Khoo J.J., AbuBakar S. (2022). Leptospirosis among dengue-negative febrile patients in Selangor, Malaysia. Am. J. Trop. Med. Hyg..

[11] Ibrahim I.N., Shimizu K., Yoshimatsu K., Yunianto A., Salwati E., Yasuda S., Koma T., Rika E., Arikawa J. (2013). Epidemiology of hantavirus infection in Thousand Islands regency of Jakarta, Indonesia. J. Vet. Med. Sci..

[12] Leibler J.H., Zakhour C.M., Gadhoke P., Gaeta J.M. (2016). Zoonotic and vector-borne infections among urban homeless and marginalized people in the United States and Europe, 1990-2014. Vector Borne Zoonotic Dis..

[13] Panthawong A., Grieco J.P., Ngoen-klan R., Chao C.C., Chareonviriyaphap T. (2020). Detection of *Anaplasma* spp. and Bartonella spp. from wild-caught rodents and their ectoparasites in Nakhon Ratchasima Province, Thailand. J. Vector Ecol..

[14] Tay S.T., Mohamed Zan H.A., Lim Y.A., Ngui R. (2013). Antibody prevalence and factors associated with exposure to *Orientia tsutsugamushi* in different aboriginal subgroups in West Malaysia. PLoS Negl. Trop. Dis..

[15] Lai W.T. (2011). Gender and livelihoods: A case study of the Mah Meri and the oil palm plantations of Carey Island. Asian J. Women’s Stud..

[16] Sinniah B., Sabaridah I., Soe M., Sabitha P., Awang I., Ong G., Hassan A. (2012). Determining the prevalence of intestinal parasites in three Orang Asli (Aborigines) communities in Perak, Malaysia. Trop. Biomed..

[17] Loong S.K., Chen H., Ling I., Nellis S., Khor C., Mohd Rahim N., Hassan H., Chao C.C., Abu Bakar S. (2018). Serological evidence of high *Leptospira* exposure among indigenous people (Orang Asli) in Peninsular Malaysia using a recombinant antigen-based ELISA. Trop. Biomed..

[18] Sheela A., Ghermandi A., Vineetha P., Sheeja R., Justus J., Ajayakrishna K. (2017). Assessment of relation of land use characteristics with vector-borne diseases in tropical areas. Land Use Policy.

[19] Varkkey H., Tyson A., Choiruzzad S.A.B. (2018). Palm oil intensification and expansion in Indonesia and Malaysia: Environmental and socio-political factors influencing policy. For. Policy Econ..

[20] Watt G., Jongsakul K., Chouriyagune C., Paris R. (2003). Differentiating dengue virus infection from scrub typhus in Thai adults with fever. Am. J. Trop. Med. Hyg..

[21] Mohd-Azami SN I., Loong S.K., Khoo J.J., Sahimin N., Lim F.S., Husin N.A., Mahfodz N.H., Mohd-Taib F.S., Ishak S.N., Makepeace B.L. (2022). Molecular evidence of rat bocavirus among rodents in Peninsular Malaysia. J. Vet. Med. Sci..

[22] Ruedas L. (2008). A guide to the mammals of Southeast Asia. Q. Rev. Biol..

[23] Herbreteau V., Jittapalapong S., Rerkamnuaychoke W., Chaval Y., Cosson J.F., Morand S. (2011). Protocols for Field and Laboratory Rodent Studies. http://www.ceropath.org/FichiersComplementaires/Herbreteau_Rodents_protocols_2011.pdf.

[24] Masakhwe C., Linsuwanon P., Kimita G., Mutai B., Leepitakrat S., Yalwala S., Abuom D., Auysawasi N., Gilbreath T., Wanja E. (2018). Identification and characterization of *Orientia chuto* in Trombiculid chigger mites collected from wild rodents in Kenya. J. Clin. Microbiol..

[25] Lau A.C., Qiu Y., Moustafa MA M., Nakao R., Shimozuru M., Onuma M., Mohd-Azlan J., Tsubota T. (2020). Detection of *Borrelia burgdorferi* Sensu Lato and relapsing fever *Borrelia* in feeding *Ixodes* ticks and rodents in Sarawak, Malaysia: New geographical records of *Borrelia yangtzensis* and *Borrelia miyamotoi*. J. Pathogens.

[26] Roux V., Rydkina E., Eremeeva M., Raoult D. (1997). Citrate synthase gene comparison, a new tool for phylogenetic analysis, and its application for the rickettsiae. Int. J. Syst. Evol. Microbiol..

[27] Labruna M.B., Whitworth T., Horta M.C., Bouyer D.H., McBride J.W., Pinter A., Popov V., Gennari S.M., Walker D.H. (2004). *Rickettsia* species infecting *Amblyomma cooperi* ticks from an area in the state of São Paulo, Brazil, where Brazilian spotted fever is endemic. J. Clin. Microbiol..

[28] Inoue K., Maruyama S., Kabeya H., Yamada N., Ohashi N., Sato Y., Yukawa M., Masuzawa T., Kawamori F., Kadosaka T. (2008). Prevalence and genetic diversity of *Bartonella* species isolated from wild rodents in Japan. Appl. Environ. Microbiol..

[29] Margos G., Gatewood A.G., Aanensen D.M., Hanincová K., Terekhova D., Vollmer S.A., Cornet M., Piesman J., Donaghy M., Bormane A. (2008). MLST of housekeeping genes captures geographic population structure and suggests a European origin of *Borrelia burgdorferi*. Proc. Natl. Acad. Sci. USA.

[30] Sonthayanon P., Peacock S.J., Chierakul W., Wuthiekanun V., Blacksell S.D., Holden MT G., Bentley S.D., Feil E.J., Day N.P.J. (2010). High rates of homologous recombination in the mite endosymbiont and opportunistic human pathogen *Orientia tsutsugamushi*. PLoS Negl. Trop. Dis..

[31] Kumar S., Stecher G., Li M., Knyaz C., Tamura K. (2018). MEGA X: Molecular Evolutionary Genetics Analysis across computing platforms. J Mol. Biol. Evol..

[32] Drummond A.J., Suchard M.A., Xie D., Rambaut A. (2012). Bayesian phylogenetics with Beauti and the Beast 1.7. Mol. Biol. Evol..

[33] Tamura K., Stecher G., Kumar S. (2021). MEGA11: Molecular Evolutionary Genetics Analysis Version 11. Mol. Biol. Evol..

[34] Rambaut A., Drummond A.J., Xie D., Baele G., Suchard M.A. (2018). Posterior summarization in bayesian phylogenetics using Tracer 1.7. Syst. Biol..

[35] Khoo J.J., Lim F.S., Tan K.K., Chen F.S., Phoon W.H., Khor C.S., Pike B.L., Chang L.Y., AbuBakar S. (2017). Detection in Malaysia of a *Borrelia* sp. from *Haemaphysalis hystricis* (Ixodida: Ixodidae). J. Med. Entomol..

[36] Khoo J.J., Ishak S.N., Lim F.S., Mohd-Taib F.S., Khor C.S., Loong S.K., AbuBakar S. (2018). Detection of a *Borrelia* sp. from *Ixodes granulatus* ticks collected from rodents in Malaysia. J. Med. Entomol..

[37] Binetruy F., Garnier S., Boulanger N., Talagrand-Reboul É., Loire E., Faivre B., Noël V., Buysse M., Duron O. (2020). A novel *Borrelia* species, intermediate between Lyme disease and relapsing fever groups, in neotropical passerine-associated ticks. Sci. Rep..

[38] Gassmann G.S., Kramer M., Göbel U.B., Wallich R. (1989). Nucleotide sequence of a gene encoding the *Borrelia burgdorferi* flagellin. Nucleic Acids Res..

[39] Park H.S., Lee J.H., Jeong E.J., Koh S.E., Park T.K., Jang W.J., Park K.H., Kim B.J., Kook Y.H., Lee S.H. (2004). Evaluation of groEL gene analysis for identification of *Borrelia burgdorferi* Sensu Lato. J. Clin. Microbiol..

[40] Elliott I., Pearson I., Dahal P., Thomas N.V., Roberts T., Newton P.N. (2019). Scrub typhus ecology: A systematic review of *Orientia* in vectors and hosts. Parasites Vectors.

[41] Tay S.T., Kaewanee S., Ho T.M., Rohani M.Y., Devi S. (1998). Serological evidence of natural infection of wild rodents (*Rattus* spp. and *Tupaia glis*) with rickettsiae in Malaysia. Southeast Asian J. Trop. Med. Public Health.

[42] Frances S.P., Watcharapichat P., Phulsuksombati D., Tanskul P. (1999). Occurrence of *Orientia tsutsugamushi* in chiggers (Acari: Trombiculidae) and small animals in an orchard near Bangkok, Thailand. J. Med. Entomol..

[43] Frances S.P., Watcharapichat P., Phulsuksombati D., Tanskul P. (2001). Investigation of the role of *Blankaartia acuscutellaris* (Acari: Trombiculidae) as a vector of scrub typhus in central Thailand. Southeast Asian J. Trop. Med. Public Health.

[44] Rodkvamtook W., Ruang-Areerate T., Gaywee J., Richards A.L., Jeamwattanalert P., Bodhidatta D., Sangjun N., Prasartvit A., Jatisatienr A., Jatisatienr C. (2011). Isolation and characterization of *Orientia tsutsugamushi* from rodents captured following a scrub typhus outbreak at a military training base, Bothong district, Chonburi province, central Thailand. Am. J. Trop. Med. Hyg..

[45] Chareonviriyaphap T., Leepitakrat W., Lerdthusnee K., Chao C.C., Ching W.M. (2014). Dual exposure of *Rickettsia typhi* and *Orientia tsutsugamushi* in the field-collected *Rattus* rodents from Thailand. J. Vector Ecol..

[46] Linsuwanon P., Krairojananan P., Rodkvamtook W., Leepitakrat S., Davidson S., Wanja E. (2018). Surveillance for scrub typhus, rickettsial diseases, and leptospirosis in US and multinational military training exercise Cobra Gold sites in Thailand. US Army Med. Dep. J..

[47] Rodkvamtook W., Kuttasingkee N., Linsuwanon P., Sudsawat Y., Richards A.L., Somsri M., Sangjun N., Chao C.C., Davidson S., Wanja E. (2018). Scrub typhus outbreak in Chonburi Province, Central Thailand, 2013. Emerg. Infect. Dis..

[48] Elders P.N.D., Swe M.M.M., Phyo A.P., McLean A.R.D., Lin H.N., Soe K., Htay W.Y.A., Tanganuchitcharnchai A., Hla T.K., Tun N.N. (2021). Serological evidence indicates widespread distribution of rickettsioses in Myanmar. Int. J. Infect. Dis..

[49] Blacksell S.D., Luksameetanasan R., Kalambaheti T., Aukkanit N., Paris D.H., McGready R., Nosten F., Peacock S.J., Day N.P.J. (2008). Genetic typing of the 56-kDa type-specific antigen gene of contemporary *Orientia tsutsugamushi* isolates causing human scrub typhus at two sites in north-eastern and western Thailand. FEMS Immunol. Med. Microbiol..

[50] Hanifah A. (2013). Detection of *Orientia tsutsugamushi* in chiggers and tissues of small mammals using polymerase chain reactions. Experiment.

[51] Wongprompitak P., Anukool W., Wongsawat E., Silpasakorn S., Duong V., Buchy P., Morand S., Frutos R., Ekpo P., Suputtamongkol Y. (2013). Broad-coverage molecular epidemiology of *Orientia tsutsugamushi* in Thailand. Infect. Genet. Evol..

[52] Elliott I., Thangnimitchok N., Chaisiri K., Wangrangsimakul T., Jaiboon P., Day N.P.J., Paris D.H., Newton P.N., Morand S. (2021). *Orientia tsutsugamushi* dynamics in vectors and hosts: Ecology and risk factors for foci of scrub typhus transmission in northern Thailand. Parasites Vectors.

[53] Tay S., Rohani M., Devi S. (2002). Isolation and PCR detection of rickettsiae from clinical and rodent samples in Malaysia. Southeast Asian J. Trop. Med. Public Health.

[54] Chaisiri K., Cosson J.-F., Morand S. (2017). Infection of rodents by *Orientia tsutsugamushi*, the agent of scrub typhus in relation to land use in Thailand. Trop. Med. Infect. Dis..

[55] Takhampunya R., Korkusol A., Promsathaporn S., Tippayachai B., Leepitakrat S., Richards A.L., Davidson S.A. (2018). Heterogeneity of *Orientia tsutsugamushi* genotypes in field-collected trombiculid mites from wild-caught small mammals in Thailand. PLoS Negl. Trop. Dis..

[56] Takhampunya R., Korkusol A., Pongpichit C., Yodin K., Rungrojn A., Chanarat N., Promsathaporn S., Monkanna T., Thaloengsok S., Tippayachai B. (2019). Metagenomic approach to characterizing disease epidemiology in a disease-endemic environment in northern Thailand. Front. Microbiol..

[57] Peterson A.C., Ghersi B.M., Alda F., Firth C., Frye M.J., Bai Y., Osikowicz L.M., Riegel C., Lipkin I., Kosoy M.Y. (2017). Rodent-borne *Bartonella* infection varies according to host species within and among cities. EcoHealth.

[58] Elisberg B., Campbell J., Bozeman F. (1968). Antigenic diversity of *Rickettsia tsutsugamushi*: Epidemiologic and ecologic significance. J. Hyg. Epidemiol. Microbiol. Immunol..

[59] Shirai A., Tanskul P., Andre R., Dohany A., Huxsoll D. (1981). *Rickettsia tsutsugamushi* strains found in chiggers collected in Thailand. Southeast Asian J. Trop. Med. Public Health.

[60] Duong V., Mai TT X., Blasdell K., Lo L.V., Morvan C., Lay S., Anukool W., Wongprompitak P., Suputtamongkol Y., Laurent D. (2013). Molecular epidemiology of *Orientia tsutsugamushi* in Cambodia and Central Vietnam reveals a broad region-wide genetic diversity. Infect. Genet. Evol..

[61] Lan Anh L.T., Viet Cuong V., Van Toan T., Thi Hong Nhung H., Van Anh L.T., Thi Thu Thuy C., Thi Ha Giang P., Thi Thanh Nga B., Thi Lan Anh B., Van Chau N. (2020). Detection of DNA of *Rickettsia* and *Orientia tsutsugamushi* in rodents and ectoparasites in Ha Giang Province. Vietnam J. Biotechnol..

[62] Hotta K., Pham HT T., Hoang H.T., Trang T.C., Vu T.N., Ung TT H., Shimizu K., Arikawa J., Yamada A., Nguyen H.T. (2016). Prevalence and phylogenetic analysis of *Orientia tsutsugamushi* in small mammals in Hanoi, Vietnam. Vector Borne Zoonotic Dis..

[63] Ernieenor FC L., NorJaiza M.J., Fadillah A., Canedy J., Mariana A. (2021). Screening and genotyping of *Orientia tsutsugamushi* from field-collected on-host chiggers (Acari: Prostigmata) recovered from a positive scrub typhus locality in Kelantan, Malaysia. Exp. Appl. Acarol..

[64] Alkathiry H., Al-Rofaai A., Ya’cob Z., Cutmore T.S., Mohd-Azami SN I., Husin N.A., Lim F.S., Koosakulnirand S., Mahfodz N.H., Ishak S.N. (2022). Habitat and season drive chigger mite diversity and abundance on small mammals in Peninsular Malaysia. Pathogens.

[65] Nasir M.H., Mispan M.S., Bhassu S., Khoo J.J., Abubakar S., Mohd-Azami S.N.I., Ishak S.N., Mohd-Taib F.S., Omar H. (2022). Spatial distribution of *Rattus* species (Rodentia: Muridae) in oil palm plantations of Peninsular Malaysia with species verification using Cytochrome Oxidase I (COI) gene. J. Oil Palm Res..

[66] Bordes F., Blasdell K., Morand S. (2015). Transmission ecology of rodent-borne diseases: New frontiers. Integr. Zool..

[67] Kosoy M., Khlyap L., Cosson J.F., Morand S. (2015). Aboriginal and invasive rats of genus *Rattus* as hosts of infectious agents. Vector Borne Zoonotic Dis..

[68] Morand S., Bordes F., Blasdell K., Pilosof S., Cornu J.-F., Chaisiri K., Chaval Y., Cosson J.-F., Claude J., Feyfant T. (2015). Assessing the distribution of disease-bearing rodents in human-modified tropical landscapes. J. Appl. Ecol..

[69] Paris D.H., Aukkanit N., Jenjaroen K., Blacksell S.D., Day N.P. (2009). A highly sensitive quantitative real-time PCR assay based on the groEL gene of contemporary Thai strains of *Orientia tsutsugamushi*. Clin. Microbiol. Infect..

[70] Jiang J., Paris D.H., Blacksell S.D., Aukkanit N., Newton P.N., Phetsouvanh R., Izzard L., Stenos J., Graves S.R., Day N.P.J. (2013). Diversity of the 47-kDa HtrA nucleic acid and translated amino acid sequences from 17 recent human isolates of *Orientia*. Vector Borne Zoonotic Dis..

[71] Tay S.T., Kamalanathan M., Suan K.A., Chun S., Ming H., Md Yasin R., Sekaran S. (1999). Seroepidemiologic survey of *Orientia tsutsugamushi*, *Rickettsia typhi*, and TT118 spotted fever group rickettsiae in rubber estate workers in Malaysia. Am. J. Trop. Med. Hyg..

[72] Tay S.T., Ho T.M., Rohani M.Y., Devi S. (2000). Antibodies to *Orientia tsutsugamushi*, *Rickettsia typhi* and spotted fever group rickettsiae among febrile patients in rural areas of Malaysia. Trans. R. Soc. Trop. Med. Hyg..

[73] Sagin D.D., Ismail G., Nasian L.M., Jok J.J., Pang E.K. (2000). Rickettsial infection in five remote Orang Ulu villages in upper Rejang River, Sarawak, Malaysia. Southeast Asian J. Trop. Med. Public Health.

[74] Furuno K., Lee K., Itoh Y., Suzuki K., Yonemitsu K., Kuwata R., Shimoda H., Watarai M., Maeda K., Takano A. (2017). Epidemiological study of relapsing fever borreliae detected in *Haemaphysalis* ticks and wild animals in the western part of Japan. PLoS ONE.

[75] Kumagai Y., Sato K., Taylor K.R., Zamoto-Niikura A., Imaoka K., Morikawa S., Ohnishi M., Kawabata H. (2018). A relapsing fever group *Borrelia* sp. is widely distributed among wild deer in Japan. Ticks Tick Borne Dis..

[76] Nakayama S., Kobayashi T., Nakamura A., Yoshitomi H., Song Y., Ashizuka Y. (2019). Detection of *Borrelia* DNA in tick species collected from vegetation and wild animals in Fukuoka, Japan. Jpn. J. Infect. Dis..

[77] Khoo J.J., Husin N.A., Lim F.S., Oslan SN H., Mohd Azami SN I., To S.W., Abd Majid M.A., Lee H.Y., Loong S.K., Khor C.S. (2021). Molecular detection of pathogens from ectoparasites recovered from peri-domestic animals, and the first description of a *Candidatus* Midichloria sp. from *Haemaphysalis wellingtoni* from rural communities in Malaysia. Parasitol. Int..

[78] Takhampunya R., Thaloengsok S., Tippayachai B., Promsathaporn S., Leepitakrat S., Gross K., Davidson S.A. (2021). Retrospective survey of *Borrelia* spp. from rodents and ticks in Thailand. J. Med. Entomol..

[79] Schwan T.G., Anderson J.M., Lopez J.E., Fischer R.J., Raffel S.J., McCoy B.N., Safronetz D., Sogoba N., Maïga O., Traoré S.F. (2012). Endemic foci of the tick-borne relapsing fever spirochete *Borrelia crocidurae* in Mali, West Africa, and the potential for human infection. PLoS Negl. Trop. Dis..

[80] Ndiaye EH I., Diouf F.S., Ndiaye M., Bassene H., Raoult D., Sokhna C., Parola P., Diatta G. (2021). Tick-borne relapsing fever borreliosis, a major public health problem overlooked in Senegal. PLoS Negl. Trop. Dis..

[81] Taylor K.R., Takano A., Konnai S., Shimozuru M., Kawabata H., Tsubota T. (2013). *Borrelia miyamotoi* infections among wild rodents show age and month independence and correlation with *Ixodes persulcatus* larval attachment in Hokkaido, Japan. Vector Borne Zoonotic Dis..

[82] Siński E., Welc-Falęciak R., Zajkowska J. (2016). *Borrelia miyamotoi*: A human tick-borne relapsing fever spirochete in Europe and its potential impact on public health. Adv. Med. Sci..

[83] Platonov A.E., Karan L.S., Kolyasnikova N.M., Makhneva N.A., Toporkova M.G., Maleev V.V., Fish D., Krause P.J. (2011). Humans infected with relapsing fever spirochete *Borrelia miyamotoi*, Russia. Emerg. Infect. Dis..

[84] Sthitmatee N., Jinawan W., Jaisan N., Tangjitjaroen W., Chailangkarn S., Sodarat C., Ekgatat M., Padungtod P. (2016). Genetic and immunological evidences of *Borrelia burgdorferi* in dog in Thailand. Southeast Asian J. Trop. Med. Public Health.

[85] Kawabata H., Takano A., Kadosaka T., Fujita H., Nitta Y., Gokuden M., Honda T., Tomida J., Kawamura Y., Masuzawa T. (2013). Multilocus sequence typing and DNA similarity analysis implicates that a *Borrelia valaisiana*-related sp. isolated in Japan is distinguishable from European *B. Valaisiana*. J. Vet. Med. Sci..

[86] Loong S.K., Ishak S.N., Lim F.S., Khoo J.J., Tan S.N., Freddy-Jalin E.J., Mohd-Taib F.S., AbuBakar S. (2018). *Paenibacillus lautus*, an opportunistic bacterial pathogen, isolated from *Ixodes granulatus* Supino (Acari: Ixodidae) collected from a Müller’s giant Sunda rat (*Sundamys muelleri*). Syst. Appl. Acarol.

[87] Margos G., Chu C.Y., Takano A., Jiang B.G., Liu W., Kurtenbach K., Masuzawa T., Fingerle V., Cao W.C., Kawabata H. (2015). *Borrelia yangtzensis* sp. nov., a rodent-associated species in Asia, is related to *Borrelia valaisiana*. Int. J. Syst. Evol. Microbiol..

[88] Kim C.M., Yun N.R., Kim D.M. (2021). Case report: The first *Borrelia yangtzensis* infection in a human in Korea. Am. J. Trop. Med. Hyg..

[89] Blasdell K.R., Perera D., Firth C. (2019). High prevalence of rodent-borne *Bartonella* spp. in urbanizing environments in Sarawak, Malaysian Borneo. Am. J. Trop. Med. Hyg..

[90] Low V., Tan T., Ibrahim J., AbuBakar S., Lim Y. (2020). First evidence of *Bartonella phoceensis* and *Candidatus* Mycoplasma haemomuris subsp. ratti in synanthropic rodents in Malaysia. Asian Pac. J. Trop. Med..

[91] Asyikha R., Sulaiman N., Mohd-Taib F.S. (2020). Detection of *Bartonella* sp. in ticks and their small mammal hosts in mangrove forests of Peninsular Malaysia. Trop. Biomed..

[92] Klangthong K., Promsthaporn S., Leepitakrat S., Schuster A.L., McCardle P.W., Kosoy M., Takhampunya R. (2015). The distribution and diversity of *Bartonella* species in rodents and their ectoparasites across Thailand. PLoS ONE.

