# Supplementary material for: Molecular Surveillance for Vector-Borne Bacteria in Rodents and Tree Shrews of Peninsular Malaysia Oil Palm Plantations
Source: Trop Med Infect Dis. 2023 Jan 19;8(2):74. doi: 10.3390/tropicalmed8020074 (PMC9965954; doi:10.3390/tropicalmed8020074)
Supplement: Supplementary file 1 [file tropicalmed-08-00074-s001.zip › tropicalmed-2117596-supplementary.pdf]

Supplementary Figures

Figure S1. Positions with heterozygous double peaks in the *sucD* allele of *Orientia tsutsugamushi*.

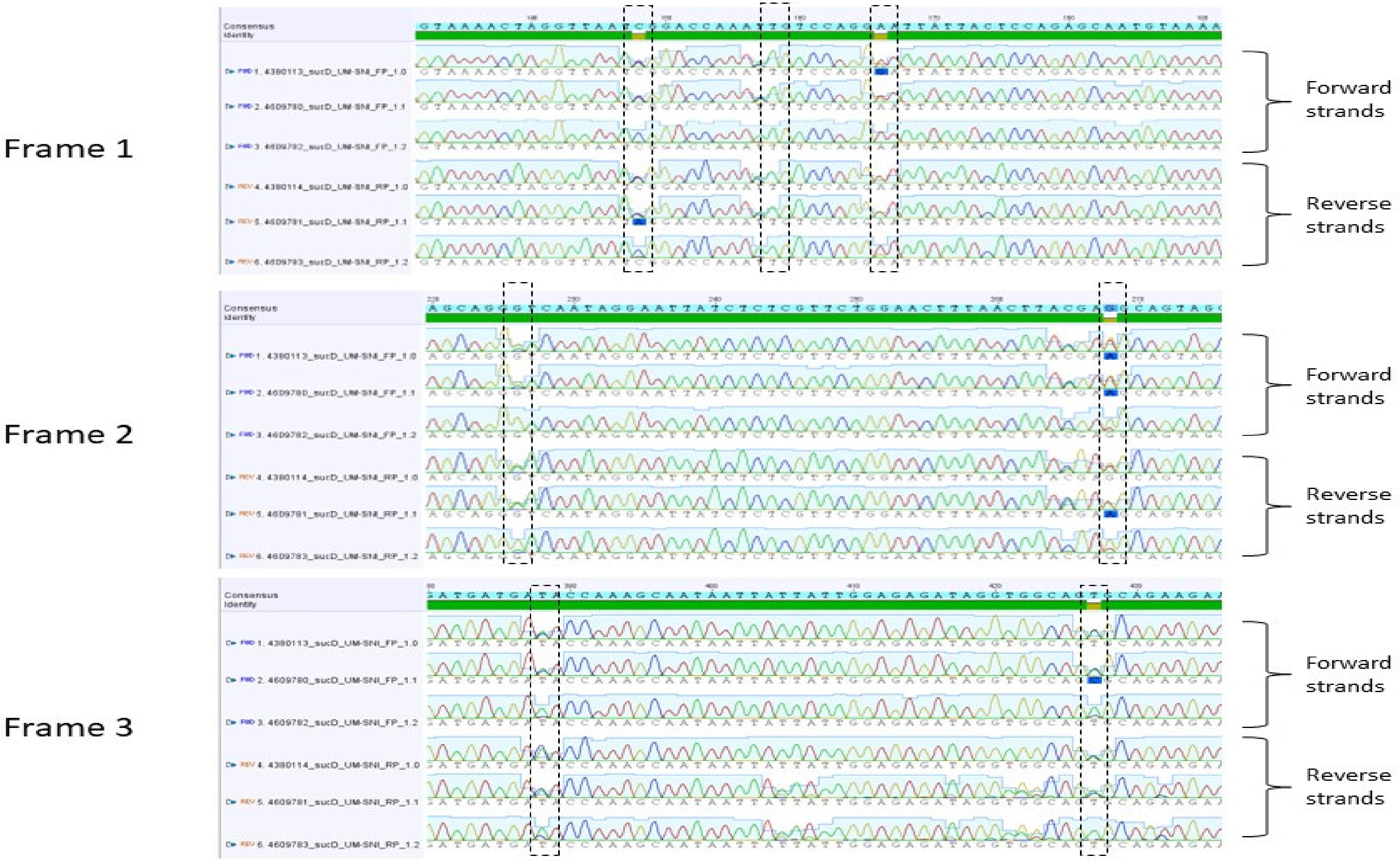

Figure S2. Positions with heterozygous double peaks in the *ppdK* allele of *Orientia tsutsugamushi*.

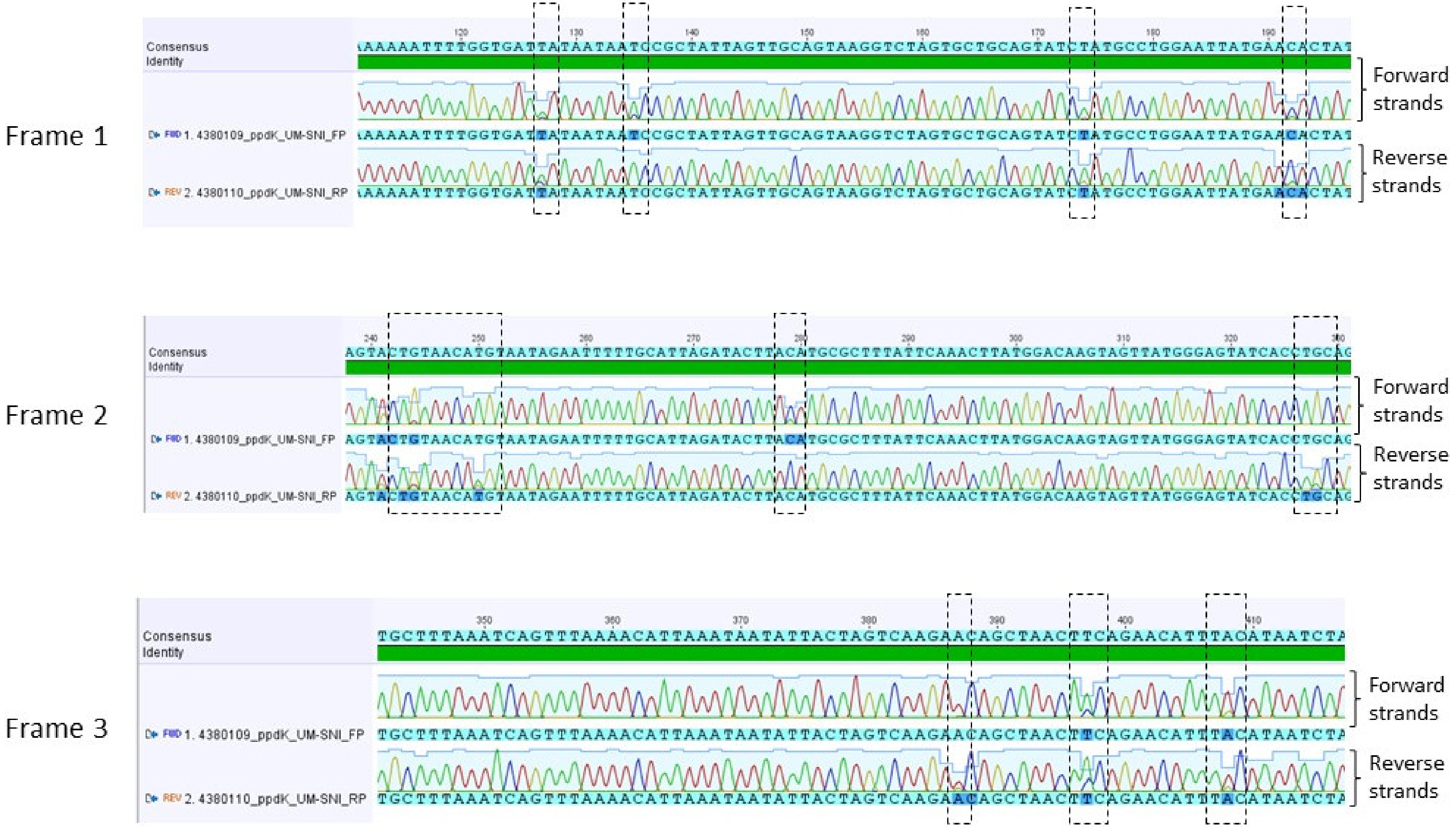

Figure S3. Similarities of *clpA* allele 310 (*Borrelia yangtzensis*) with closely related *clpA* alleles in the PubMLST database.

Allele	% Identity	Mismatches	Gaps	Alignment	Compare
clpA: 82	99.136	5	0	579/579	
clpA: 81	99.136	5	0	579/579	
clpA: 76	98.964	6	0	579/579	
clpA: 100	98.791	7	0	579/579	
clpA: 79	98.618	8	0	579/579	
clpA: 78	98.618	8	0	579/579	
clpA: 83	98.273	10	0	579/579	
clpA: 89	98.100	11	0	579/579	
clpA: 84	98.100	11	0	579/579	
clpA: 75	98.100	11	0	579/579	
clpA: 80	97.582	14	0	579/579	
clpA: 50	96.718	19	0	579/579	
clpA: 257	96.718	19	0	579/579	
clpA: 49	96.546	20	0	579/579	
clpA: 110	96.546	20	0	579/579	
clpA: 96	96.373	21	0	579/579	
clpA: 77	96.200	22	0	579/579	
clpA: 297	94.646	31	0	579/579	
clpA: 179	91.883	47	0	579/579	
clpA: 32	91.537	49	0	579/579	
clpA: 299	91.537	49	0	579/579	
clpA: 234	91.537	49	0	579/579	
clpA: 74	91.379	48	2	580/579	
clpA: 74	91.379	48	2	580/579	
clpA: 28	91.364	50	0	579/579	
clpA: 26	91.364	50	0	579/579	

Figure S4. *Borrelia yangtzensis* ST360 strains deposited in the PubMLST database.

Isolate fields							MLST								
id	isolate	aliases	country	species	year	source	clpA	clpX	nifS	pepX	pyrG	recG	rplB	uvrA	ST
1262	Okinawa-MC8B05		Japan	Borrelia yangtzensis		animal host	81	66	59	70	74	62	58	58	360
1272	066-5		Japan	Borrelia yangtzensis		tick	81	66	59	70	74	62	58	58	360

Supplementary Figure 5. The unique gap in the *flaB* sequences of *Borrelia* sp. strain UM-SNI19 (red box), compared to other borreliae strains.

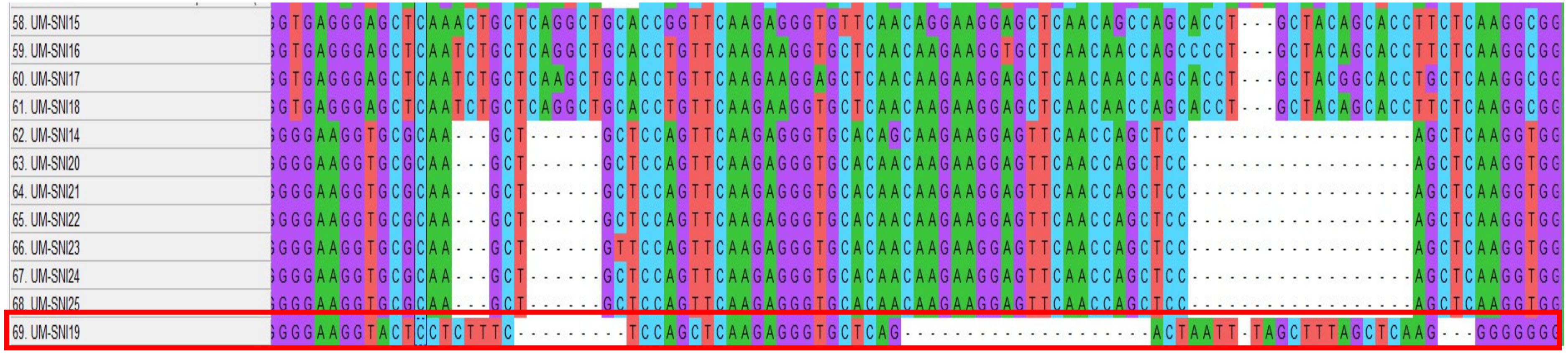

Supplementary Table

Table S1. The morphological information of small mammals trapped in Johor and Perak, Malaysia.

No.	Species	Voucher No.	Habitat	Locality	Sex	Age	Microorganism detected	Strain	Accession No.	Total number of individuals
1.	*Rattus tanezumi* R3 mitotype	KTR003	Oil palm plantation	Johor, Malaysia	Female	Sub-adult	-	-	-	113
2.	*Rattus tanezumi* R3 mitotype	KTR004	Oil palm plantation	Johor, Malaysia	Male	Adult	-	-	-	
3.	*Rattus tanezumi* R3 mitotype	KTR005	Oil palm plantation	Johor, Malaysia	Female	Sub-adult	-	-	-	
4.	*Rattus tanezumi* R3 mitotype	KTR010	Oil palm plantation	Johor, Malaysia	Male	Sub-adult	*Orientia tsutsugamushi*	UM-SNI41	OP413418	
5.	*Rattus tanezumi* R3 mitotype	KTR011	Oil palm plantation	Johor, Malaysia	Female	Adult	-	-	-	
6.	*Rattus tanezumi* R3 mitotype	KTR013	Oil palm plantation	Johor, Malaysia	Female	Juvenile	-	-	-	
7.	*Rattus tanezumi* R3 mitotype	KTR021	Oil palm plantation	Johor, Malaysia	Female	Sub-adult	-	-	-	
8.	*Rattus tanezumi* R3 mitotype	KTR022	Oil palm plantation	Johor, Malaysia	Male	Sub-adult	-	-	-	
9.	*Rattus tanezumi* R3 mitotype	KTR026	Oil palm plantation	Johor, Malaysia	Female	Adult	-	-	-	
10.	*Rattus tanezumi* R3 mitotype	KTR027	Oil palm plantation	Johor, Malaysia	Female	Adult	-	-	-	
11.	*Rattus tanezumi* R3 mitotype	KTR028	Oil palm plantation	Johor, Malaysia	Female	Sub-adult	*Orientia tsutsugamushi*	UM-SNI42	OP413419	
12.	*Rattus tanezumi* R3 mitotype	KTR032	Oil palm plantation	Johor, Malaysia	Male	Adult	-	-	-	
13.	*Rattus tanezumi* R3 mitotype	KTR033	Oil palm plantation	Johor, Malaysia	Female	Sub-adult	-	-	-	

13.	*Rattus tanezumi* R3 mitotype	KTR033	Oil palm plantation	Johor, Malaysia	Female	Sub-adult	-	-	-
14.	*Rattus tanezumi* R3 mitotype	KTR034	Oil palm plantation	Johor, Malaysia	Female	Sub-adult	*Orientia tsutsugamushi*	UM-SNI43	OP413420
15.	*Rattus tanezumi* R3 mitotype	KTR035	Oil palm plantation	Johor, Malaysia	Male	Adult	-	-	-
16.	*Rattus tanezumi* R3 mitotype	KTR037	Oil palm plantation	Johor, Malaysia	Male	Adult	*Borrelia* sp. (LD)	UM-SNI16	OP375130
17.	*Rattus tanezumi* R3 mitotype	KTR041	Oil palm plantation	Johor, Malaysia	Female	Adult	*Orientia tsutsugamushi*	UM-SNI46	OP413423
18.	*Rattus tanezumi* R3 mitotype	KTR042	Oil palm plantation	Johor, Malaysia	Male	Adult	-	-	-
19.	*Rattus tanezumi* R3 mitotype	KTR043	Oil palm plantation	Johor, Malaysia	Male	Adult	*Bartonella phoceensis*/ *Orientia tsutsugamushi*	UM-SNI04/ UM-SNI47	OP375118/ OP413424
20.	*Rattus tanezumi* R3 mitotype	KTR044	Oil palm plantation	Johor, Malaysia	Female	Adult	*Borrelia* sp. (LD)	UM-SNI17	OP375131
21.	*Rattus tanezumi* R3 mitotype	KTR045	Oil palm plantation	Johor, Malaysia	Female	Adult	*Orientia tsutsugamushi*	UM-SNI48	OP413425
22.	*Rattus tanezumi* R3 mitotype	KTR058	Oil palm plantation	Johor, Malaysia	Male	Sub-adult	-	-	-
23.	*Rattus tanezumi* R3 mitotype	KTR059	Oil palm plantation	Johor, Malaysia	Male	Adult	-	-	-
24.	*Rattus tanezumi* R3 mitotype	KTR060	Oil palm plantation	Johor, Malaysia	Male	Adult	-	-	-
25.	*Rattus tanezumi* R3 mitotype	KTR061	Oil palm plantation	Johor, Malaysia	Female	Adult	-	-	-
26.	*Rattus tanezumi* R3 mitotype	KTR063	Oil palm plantation	Johor, Malaysia	Male	Juvenile	-	-	-
27.	*Rattus tanezumi* R3 mitotype	KTR068	Oil palm plantation	Johor, Malaysia	Female	Adult	-	-	-

28.	*Rattus tanezumi* R3 mitotype	KTR069	Oil palm plantation	Johor, Malaysia	Male	Adult	*Borrelia* sp. (LD)	UM-SNI18	OP375132
29.	*Rattus tanezumi* R3 mitotype	KTR070	Oil palm plantation	Johor, Malaysia	Female	Adult	-	-	-
30.	*Rattus tanezumi* R3 mitotype	KTR071	Oil palm plantation	Johor, Malaysia	Female	Sub-adult	*Orientia tsutsugamushi*	UM-SNI50	OP413427
31.	*Rattus tanezumi* R3 mitotype	KTR072	Oil palm plantation	Johor, Malaysia	Female	Adult	-	-	-
32.	*Rattus tanezumi* R3 mitotype	KTR073	Oil palm plantation	Johor, Malaysia	Male	Juvenile	-	-	-
33.	*Rattus tanezumi* R3 mitotype	KTR074	Oil palm plantation	Johor, Malaysia	Female	Juvenile	-	-	-
34.	*Rattus tanezumi* R3 mitotype	KTR075	Oil palm plantation	Johor, Malaysia	Male	Juvenile	-	-	-
35.	*Rattus tanezumi* R3 mitotype	KTR077	Oil palm plantation	Johor, Malaysia	Male	Juvenile	-	-	-
36.	*Rattus tanezumi* R3 mitotype	KTR078	Oil palm plantation	Johor, Malaysia	Male	Sub-adult	-	-	-
37.	*Rattus tanezumi* R3 mitotype	KTR079	Oil palm plantation	Johor, Malaysia	Female	Adult	-	-	-
38.	*Rattus tanezumi* R3 mitotype	KTR080	Oil palm plantation	Johor, Malaysia	Female	Adult	-	-	-
39.	*Rattus tanezumi* R3 mitotype	KTR081	Oil palm plantation	Johor, Malaysia	Female	Adult	-	-	-
40.	*Rattus tanezumi* R3 mitotype	KTR082	Oil palm plantation	Johor, Malaysia	Female	Adult	-	-	-
41.	*Rattus tanezumi* R3 mitotype	KTR083	Oil palm plantation	Johor, Malaysia	Female	Adult	-	-	-
42.	*Rattus tanezumi* R3 mitotype	KTR086	Oil palm plantation	Johor, Malaysia	Female	Adult	-	-	-
43.	*Rattus tanezumi* R3 mitotype	KTR087	Oil palm plantation	Johor, Malaysia	Male	Adult	-	-	-

44.	*Rattus tanezumi* R3 mitotype	KTR093	Oil palm plantation	Johor, Malaysia	Male	Adult	-	-	-
45.	*Rattus tanezumi* R3 mitotype	KTR094	Oil palm plantation	Johor, Malaysia	Female	Adult	-	-	-
46.	*Rattus tanezumi* R3 mitotype	KTR095	Oil palm plantation	Johor, Malaysia	Female	Adult	-	-	-
47.	*Rattus tanezumi* R3 mitotype	KTR096	Oil palm plantation	Johor, Malaysia	Male	Adult	-	-	-
48.	*Rattus tanezumi* R3 mitotype	KTR097	Oil palm plantation	Johor, Malaysia	Male	Sub-adult	-	-	-
49.	*Rattus tanezumi* R3 mitotype	KTR098	Oil palm plantation	Johor, Malaysia	Female	Adult	-	-	-
50.	*Rattus tanezumi* R3 mitotype	KTR099	Oil palm plantation	Johor, Malaysia	Male	Juvenile	-	-	-
51.	*Rattus tanezumi* R3 mitotype	KTR101	Oil palm plantation	Johor, Malaysia	Female	Juvenile	-	-	-
52.	*Rattus tanezumi* R3 mitotype	THTR003	Paddy field	Perak, Malaysia	Male	-	-	-	-
53.	*Rattus tanezumi* R3 mitotype	THTR004	Oil palm plantation	Perak, Malaysia	Female	Adult	*Bartonella phoceensis*	UM-SNI05	OP375119
54.	*Rattus tanezumi* R3 mitotype	THTR005	Oil palm plantation	Perak, Malaysia	Female	Adult	-	-	-
55.	*Rattus tanezumi* R3 mitotype	THTR006	Residential area	Perak, Malaysia	Male	Adult	*Bartonella phoceensis*	UM-SNI06	OP375120
56.	*Rattus tanezumi* R3 mitotype	THTR007	Residential area	Perak, Malaysia	Male	Sub-adult	*Bartonella phoceensis*	UM-SNI07	OP375121
57.	*Rattus tanezumi* R3 mitotype	THTR008	Residential area	Perak, Malaysia	Female	Adult	*Bartonella phoceensis*	UM-SNI08	OP375122
58.	*Rattus tanezumi* R3 mitotype	THTR009	Residential area	Perak, Malaysia	Female	Sub-adult	-	-	-
59.	*Rattus tanezumi* R3 mitotype	THTR010	Residential area	Perak, Malaysia	Female	Adult	*Bartonella phoceensis/*	UM-SNI09/ UM-SNI26	OP375122/ OP413403

							Orientia tsutsugamushi		
60.	*Rattus tanezumi* R3 mitotype	THTR013	Residential area	Perak, Malaysia	Female	Adult	-	-	-
61.	*Rattus tanezumi* R3 mitotype	THTR015	Oil palm plantation	Perak, Malaysia	Male	Adult	-	-	-
62.	*Rattus tanezumi* R3 mitotype	THTR016	Oil palm plantation	Perak, Malaysia	Male	Adult	-	-	-
63.	*Rattus tanezumi* R3 mitotype	THTR019	Oil palm plantation	Perak, Malaysia	Male	Adult	-	-	-
64.	*Rattus tanezumi R3 mitotype*	THTR042	Oil palm plantation	Perak, Malaysia	Female	Adult	*Orientia tsutsugamushi*	UM-SNI29	OP413406
65.	*Rattus tanezumi R3 mitotype*	THTR043	Oil palm plantation	Perak, Malaysia	Female	Adult	*Bartonella phoceensis*/ *Orientia tsutsugamushi*	UM-SNI11/ UM-SNI30	OP375125/ OP413407
66.	*Rattus tanezumi R3 mitotype*	THTR044	Oil palm plantation	Perak, Malaysia	Female	Adult	-	-	-
67.	*Rattus tanezumi* R3 mitotype	THTR046	Oil palm plantation	Perak, Malaysia	Male	Sub-adult	*Bartonella phoceensis*/ *Orientia tsutsugamushi*	UM-SNI12/ UM-SNI33	OP375126/ OP413410
68.	*Rattus tanezumi* R3 mitotype	THTR047	Oil palm plantation	Perak, Malaysia	Male	Adult	*Orientia tsutsugamushi*	UM-SNI34	OP413411
69.	*Rattus tanezumi* R3 mitotype	THTR048	Oil palm plantation	Perak, Malaysia	Female	Sub-adult	*Bartonella phoceensis*/ *Orientia tsutsugamushi*	UM-SNI13/ UM-SNI35	OP375127/ OP413412
70.	*Rattus tanezumi R3 mitotype*	THTR110	Residential area	Perak, Malaysia	Male	Adult	-	-	-
71.	*Rattus tanezumi* R3 mitotype	THTR112	Oil palm plantation	Perak, Malaysia	Male	Adult	-	-	-

72.	*Rattus tanezumi R3 mitotype*	THTR113	Oil palm plantation	Perak, Malaysia	Female	Sub-adult	-	-	-
73.	*Rattus tanezumi* R3 mitotype	THTR114	Oil palm plantation	Perak, Malaysia	Female	Adult	-	-	-
74.	*Rattus tanezumi* R3 mitotype	THTR115	Oil palm plantation	Perak, Malaysia	Male	Adult	-	-	-
75.	*Rattus tanezumi* R3 mitotype	THTR118	Oil palm plantation	Perak, Malaysia	Male	Adult	-	-	-
76.	*Rattus tanezumi* R3 mitotype	THTR119	Oil palm plantation	Perak, Malaysia	Male	Juvenile	-	-	-
77.	*Rattus tanezumi* R3 mitotype	THTR120	Oil palm plantation	Perak, Malaysia	Male	Adult	*Borrelia* sp. (RF)	UM-SNI21	OP375135
78.	*Rattus tanezumi* R3 mitotype	THTR121	Oil palm plantation	Perak, Malaysia	Female	Adult	-	-	-
79.	*Rattus tanezumi* R3 mitotype	THTR123	Paddy field	Perak, Malaysia	Female	Sub-adult	-	-	-
80.	*Rattus tanezumi* R3 mitotype	THTR125	Oil palm plantation	Perak, Malaysia	Male	Sub-adult	-	-	-
81.	*Rattus tanezumi* R3 mitotype	THTR126	Oil palm plantation	Perak, Malaysia	Female	Adult	-	-	-
82.	*Rattus tanezumi* R3 mitotype	THTR127	Oil palm plantation	Perak, Malaysia	Female	Adult	-	-	-
83.	*Rattus tanezumi* R3 mitotype	THTR128	Oil palm plantation	Perak, Malaysia	Male	Adult	*Borrelia* sp. (RF)	UM-SNI22	OP375136
84.	*Rattus tanezumi* R3 mitotype	THTR129	Oil palm plantation	Perak, Malaysia	Male	Juvenile	-	-	-
85.	*Rattus tanezumi* R3 mitotype	THTR133	Oil palm plantation	Perak, Malaysia	Male	Adult	*Orientia tsutsugamushi*	UM-SNI36	OP413413
86.	*Rattus tanezumi* R3 mitotype	THTR134	Oil palm plantation	Perak, Malaysia	Female	Adult	-	-	-
87.	*Rattus tanezumi* R3 mitotype	THTR136	Residential area	Perak, Malaysia	Female	Sub-adult	-	-	-

88.	*Rattus tanezumi* R3 mitotype	THTR138	Residential area	Perak, Malaysia	Male	Adult	-	-	-
89.	*Rattus tanezumi* R3 mitotype	THTR139	Residential area	Perak, Malaysia	Female	Adult	-	-	-
90.	*Rattus tanezumi* R3 mitotype	THTR140	Oil palm plantation	Perak, Malaysia	Female	Adult	-	-	-
91.	*Rattus tanezumi* R3 mitotype	THTR141	Oil palm plantation	Perak, Malaysia	Female	Adult	-	-	-
92.	*Rattus tanezumi* R3 mitotype	THTR142	Residential area	Perak, Malaysia	Male	Adult	*Borrelia burgdorferi*	UM-SNI15	OP375129
93.	*Rattus tanezumi* R3 mitotype	THTR143	Residential area	Perak, Malaysia	Male	Adult	-	-	-
94.	*Rattus tanezumi* R3 mitotype	THTR145	Oil palm plantation	Perak, Malaysia	Male	Adult	-	-	-
95.	*Rattus tanezumi* R3 mitotype	THTR146	Oil palm plantation	Perak, Malaysia	Female	Adult	-	-	-
96.	*Rattus tanezumi* R3 mitotype	THTR147	Oil palm plantation	Perak, Malaysia	Male	Adult	-	-	-
97.	*Rattus tanezumi* R3 mitotype	THTR148	Oil palm plantation	Perak, Malaysia	Male	Adult	-	-	-
98.	*Rattus tanezumi* R3 mitotype	THTR149	Oil palm plantation	Perak, Malaysia	Female	Adult	-	-	-
99.	*Rattus tanezumi* R3 mitotype	THTR153	Oil palm plantation	Perak, Malaysia	Male	Juvenile	-	-	-
100.	*Rattus tanezumi* R3 mitotype	THTR157	Residential area	Perak, Malaysia	Male	Adult	-	-	-
101.	*Rattus tanezumi* R3 mitotype	THTR158	Residential area	Perak, Malaysia	Male	Sub-adult	-	-	-
102.	*Rattus tanezumi* R3 mitotype	THTR159	Oil palm plantation	Perak, Malaysia	Female	Sub-adult	-	-	-
103.	*Rattus tanezumi* R3 mitotype	THTR160	Oil palm plantation	Perak, Malaysia	Female	Adult	-	-	-

104.	*Rattus tanezumi* R3 mitotype	THTR161	Oil palm plantation	Perak, Malaysia	Female	Adult	-	-	-
105.	*Rattus tanezumi* R3 mitotype	THTR162	Oil palm plantation	Perak, Malaysia	Female	Adult	*Borrelia* sp. (RF)	UM-SNI24	OP537179
106.	*Rattus tanezumi* R3 mitotype	THTR163	Oil palm plantation	Perak, Malaysia	Male	Adult	-	-	-
107.	*Rattus tanezumi* R3 mitotype	THTR164	Oil palm plantation	Perak, Malaysia	Female	Adult	*Orientia tsutsugamushi*	UM-SNI38	OP413415
108.	*Rattus tanezumi* R3 mitotype	THTR165	Oil palm plantation	Perak, Malaysia	Female	Sub-adult	-	-	-
109.	*Rattus tanezumi* R3 mitotype	THTR167	Oil palm plantation	Perak, Malaysia	Female	Adult	-	-	-
110.	*Rattus tanezumi* R3 mitotype	THTR168	Oil palm plantation	Perak, Malaysia	Male	Adult	-	-	-
111.	*Rattus tanezumi* R3 mitotype	THTR170	Oil palm plantation	Perak, Malaysia	Female	Adult	*Orientia tsutsugamushi*	UM-SNI39	OP413416
112.	*Rattus tanezumi* R3 mitotype	THTR171	Oil palm plantation	Perak, Malaysia	Male	Adult	*Orientia tsutsugamushi*	UM-SNI40	OP413417
113.	*Rattus tanezumi* R3 mitotype	THTR172	Oil palm plantation	Perak, Malaysia	Male	Adult	-	-	-
114.	*Rattus tanezumi* R3 mitotype	THTR173	Oil palm plantation	Perak, Malaysia	Male	Adult	-	-	-
115.	*Rattus tanezumi* R3 mitotype	THTR174	Oil palm plantation	Perak, Malaysia	Female	Sub-adult	*Borrelia* sp. (RF)	UM-SNI25	OP375138
116.	*Rattus argentiventer*	THTR001	Paddy field	Perak, Malaysia	Female	Sub-adult	-	-	-
117.	*Rattus argentiventer*	THTR020	Paddy field	Perak, Malaysia	Female	Sub-adult	-	-	-
118.	*Rattus argentiventer*	THTR021	Paddy field	Perak, Malaysia	Female	Sub-adult	-	-	-
119.	*Rattus argentiventer*	THTR022	Paddy field	Perak, Malaysia	Male	Adult	*Bartonella phoceensis*	UM-SNI10	OP375124

120.	*Rattus argentiventer*	THTR023	Paddy field	Perak, Malaysia	Male	Adult	-	-	-
121.	*Rattus argentiventer*	THTR025	Paddy field	Perak, Malaysia	Female	Sub-adult	-	-	-
122.	*Rattus argentiventer*	THTR026	Paddy field	Perak, Malaysia	Male	Sub-adult	-	-	-
123.	*Rattus argentiventer*	THTR027	Paddy field	Perak, Malaysia	Female	Adult	-	-	-
124.	*Rattus argentiventer*	THTR028	Paddy field	Perak, Malaysia	Female	Sub-adult	-	-	-
125.	*Rattus argentiventer*	THTR029	Paddy field	Perak, Malaysia	Female	Sub-adult	-	-	-
126.	*Rattus argentiventer*	THTR030	Paddy field	Perak, Malaysia	Male	Adult	-	-	-
127.	*Rattus argentiventer*	THTR031	Paddy field	Perak, Malaysia	Male	Adult	-	-	-
128.	*Rattus argentiventer*	THTR032	Paddy field	Perak, Malaysia	Female	Sub-adult	-	-	-
129.	*Rattus argentiventer*	THTR033	Paddy field	Perak, Malaysia	Male	Adult	-	-	-
130.	*Rattus argentiventer*	THTR034	Paddy field	Perak, Malaysia	Female	Adult	-	-	-
131.	*Rattus argentiventer*	THTR035	Paddy field	Perak, Malaysia	Female	Sub-adult	-	-	-
132.	*Rattus argentiventer*	THTR036	Paddy field	Perak, Malaysia	Female	Adult	*Orientia tsutsugamushi*	UM-SNI27	OP413404
133.	*Rattus argentiventer*	THTR037	Oil palm plantation	Perak, Malaysia	Male	Adult	-	-	-
134.	*Rattus argentiventer*	THTR038	Oil palm plantation	Perak, Malaysia	Female	Adult	-	-	-
135.	*Rattus argentiventer*	THTR039	Oil palm plantation	Perak, Malaysia	Female	Sub-adult	-	-	-

136.	*Rattus argentiventer*	THTR107	Paddy field	Perak, Malaysia	Male	Adult	-	-	-
137.	*Rattus argentiventer*	THTR122	Paddy field	Perak, Malaysia	Male	Sub-adult	-	-	-
138.	*Rattus argentiventer*	THTR124	Paddy field	Perak, Malaysia	Male	Adult	-	-	-
139.	*Rattus argentiventer*	THTR155	Paddy field	Perak, Malaysia	Male	Adult	-	-	-
140.	*Rattus argentiventer*	THTR156	Paddy field	Perak, Malaysia	Male	Adult	*Orientia tsutsugamushi*	UM-SNI37	OP413414
141.	*Rattus tiomanicus*	KTR001	Oil palm plantation	Johor, Malaysia	Female	Adult	-	-	-
142.	*Rattus tiomanicus*	KTR002	Oil palm plantation	Johor, Malaysia	Male	-	-	-	-
143.	*Rattus tiomanicus*	KTR006	Oil palm plantation	Johor, Malaysia	Male	Juvenile	-	-	-
144.	*Rattus tiomanicus*	KTR012	Oil palm plantation	Johor, Malaysia	Female	Adult	-	-	-
145.	*Rattus tiomanicus*	KTR023	Oil palm plantation	Johor, Malaysia	Female	Juvenile	-	-	-
146.	*Rattus tiomanicus*	KTR024	Oil palm plantation	Johor, Malaysia	Male	Adult	-	-	-
147.	*Rattus tiomanicus*	KTR029	Oil palm plantation	Johor, Malaysia	Male	Juvenile	-	-	-
148.	*Rattus tiomanicus*	KTR036	Oil palm plantation	Johor, Malaysia	Female	Adult	*Orientia tsutsugamushi*	UM-SNI44	OP413421
149.	*Rattus tiomanicus*	KTR046	Oil palm plantation	Johor, Malaysia	Male	Juvenile	-	-	-
150.	*Rattus tiomanicus*	KTR062	Oil palm plantation	Johor, Malaysia	Female	Adult	-	-	-
151.	*Rattus tiomanicus*	KTR065	Oil palm plantation	Johor, Malaysia	Male	Juvenile	-	-	-

152.	*Rattus tiomanicus*	KTR076	Oil palm plantation	Johor, Malaysia	Male	Juvenile	-	-	-
153.	*Rattus tiomanicus*	THTR012	Residential area	Perak, Malaysia	Female	Adult	-	-	-
154.	*Rattus tiomanicus*	THTR017	Oil palm plantation	Perak, Malaysia	Female	Juvenile	-	-	-
155.	*Rattus tiomanicus*	THTR018	Oil palm plantation	Perak, Malaysia	Female	Sub-adult	-	-	-
156.	*Rattus tiomanicus*	THTR040	Residential area	Perak, Malaysia	Female	Adult	-	-	-
157.	*Rattus tiomanicus*	THTR116	Oil palm plantation	Perak, Malaysia	Female	Sub-adult	-	-	-
158.	*Rattus tiomanicus*	THTR117	Oil palm plantation	Perak, Malaysia	Male	Juvenile	-	-	-
159.	*Rattus tiomanicus*	THTR137	Residential area	Perak, Malaysia	Male	Adult	-	-	-
160.	*Rattus tiomanicus*	THTR150	Oil palm plantation	Perak, Malaysia	Female	Adult	*Borrelia* sp. (RF)	UM-SNI23	OP375137
161.	*Rattus tiomanicus*	THTR166	Oil palm plantation	Perak, Malaysia	Female	Adult	-	-	-
162.	*Rattus tiomanicus*	THTR175	Oil palm plantation	Perak, Malaysia	Female	Juvenile	-	-	-
163.	*Rattus exulans*	KTR007	Oil palm plantation	Johor, Malaysia	Female	Adult	-	-	-
164.	*Rattus exulans*	KTR008	Oil palm plantation	Johor, Malaysia	Female	Adult	-	-	-
165.	*Rattus exulans*	KTR064	Oil palm plantation	Johor, Malaysia			-	-	-
166.	*Rattus exulans*	THTR002	Paddy field	Perak, Malaysia	Male	Adult	-	-	-
167.	*Rattus exulans*	THTR014	Residential area	Perak, Malaysia	Male	Juvenile	-	-	-

168.	*Rattus exulans*	THTR024	Paddy field	Perak, Malaysia	Male	Adult	-	-	-	
169.	*Rattus exulans*	THTR041	Oil palm plantation	Perak, Malaysia	Male	Adult	*Orientia tsutsugamushi*	UM-SNI28	OP413405	
170.	*Rattus exulans*	THTR045	Oil palm plantation	Perak, Malaysia	Male	Adult	-	-	-	
171.	*Rattus exulans*	THTR109	Residential area	Perak, Malaysia	Male	Adult	-	-	-	
172.	*Rattus exulans*	THTR131	Oil palm plantation	Perak, Malaysia	Female	Adult	-	-	-	
173.	*Rattus exulans*	THTR152	Oil palm plantation	Perak, Malaysia	Male	Sub-adult	-	-	-	
174.	*Rattus exulans*	THTR169	Oil palm plantation	Perak, Malaysia	Male	Juvenile	-	-	-	
175.	*Rattus exulans*	THTR176	Oil palm plantation	Perak, Malaysia	Female	-	-	-	-	
176.	*Rattus tanezumi sensu stricto*	THTR108	Paddy field	Perak, Malaysia	Female	Adult	-	-	-	1
177.	*Tupaia glis*	THTR011	Residential area	Perak, Malaysia	Female	Adult	-	-	-	40
178.	*Tupaia glis*	THTR111	Residential area	Perak, Malaysia	Female	Adult	*Borrelia* sp. (RF)	UM-SNI20	OP375134	
179.	*Tupaia glis*	THTR130	Oil palm plantation	Perak, Malaysia	Male	Adult	-	-	-	
180.	*Tupaia glis*	THTR132	Oil palm plantation	Perak, Malaysia	Male	Adult	-	-	-	
181.	*Tupaia glis*	THTR135	Residential area	Perak, Malaysia	Male	Adult	-	-	-	
182.	*Tupaia glis*	THTR144	Residential area	Perak, Malaysia	Male	Adult	-	-	-	
183.	*Tupaia glis*	THTR151	Oil palm plantation	Perak, Malaysia	Female	Adult	-	-	-	

184.	*Tupaia glis*	KTR009	Oil palm plantation	Johor, Malaysia	Female	Adult	-	-	-
185.	*Tupaia glis*	KTR014	Oil palm plantation	Johor, Malaysia	Female	Adult	-	-	-
186.	*Tupaia glis*	KTR015	Oil palm plantation	Johor, Malaysia	Female	Adult	-	-	-
187.	*Tupaia glis*	KTR016	Oil palm plantation	Johor, Malaysia	Male	Adult	-	-	-
188.	*Tupaia glis*	KTR017	Oil palm plantation	Johor, Malaysia	Female	Adult	-	-	-
189.	*Tupaia glis*	KTR018	Oil palm plantation	Johor, Malaysia	Male	Adult	-	-	-
190.	*Tupaia glis*	KTR019	Oil palm plantation	Johor, Malaysia	Male	Adult	-	-	-
191.	*Tupaia glis*	KTR020	Oil palm plantation	Johor, Malaysia	Male	Sub-adult	-	-	-
192.	*Tupaia glis*	KTR025	Oil palm plantation	Johor, Malaysia	Male	Adult	-	-	-
193.	*Tupaia glis*	KTR030	Oil palm plantation	Johor, Malaysia	Male	Adult	-	-	-
194.	*Tupaia glis*	KTR031	Oil palm plantation	Johor, Malaysia	Female	Adult	-	-	-
195.	*Tupaia glis*	KTR038	Oil palm plantation	Johor, Malaysia	Male	Adult	-	-	-
196.	*Tupaia glis*	KTR039	Oil palm plantation	Johor, Malaysia	Female	Adult	*Orientia tsutsugamushi*	UM-SNI45	OP413422
197.	*Tupaia glis*	KTR040	Oil palm plantation	Johor, Malaysia	Male	Adult	-	-	-
198.	*Tupaia glis*	KTR047	Oil palm plantation	Johor, Malaysia	Female	Adult	-	-	-
199.	*Tupaia glis*	KTR048	Oil palm plantation	Johor, Malaysia	Male	Adult	-	-	-

200.	*Tupaia glis*	KTR049	Oil palm plantation	Johor, Malaysia	Female	Adult	-	-	-
201.	*Tupaia glis*	KTR050	Oil palm plantation	Johor, Malaysia	Female	Adult	-	-	-
202.	*Tupaia glis*	KTR051	Oil palm plantation	Johor, Malaysia	Female	Adult	-	-	-
203.	*Tupaia glis*	KTR052	Oil palm plantation	Johor, Malaysia	Male	Adult	*Orientia tsutsugamushi*	UM-SNI49	OP413426
204.	*Tupaia glis*	KTR053	Oil palm plantation	Johor, Malaysia	Male	Adult	-	-	-
205.	*Tupaia glis*	KTR054	Oil palm plantation	Johor, Malaysia	Male	Adult	-	-	-
206.	*Tupaia glis*	KTR055	Oil palm plantation	Johor, Malaysia	Male	Adult	-	-	-
207.	*Tupaia glis*	KTR056	Oil palm plantation	Johor, Malaysia	Male	Adult	-	-	-
208.	*Tupaia glis*	KTR057	Oil palm plantation	Johor, Malaysia	Female	Juvenile			-
209.	*Tupaia glis*	KTR066	Oil palm plantation	Johor, Malaysia	Male	Sub-adult	-	-	-
210.	*Tupaia glis*	KTR067	Oil palm plantation	Johor, Malaysia	Male	Adult	-	-	-
211.	*Tupaia glis*	KTR084	Oil palm plantation	Johor, Malaysia	Male	Adult	-	-	-
212.	*Tupaia glis*	KTR085	Oil palm plantation	Johor, Malaysia	Female	Adult	-	-	-
213.	*Tupaia glis*	KTR088	Oil palm plantation	Johor, Malaysia	Male	Adult	-	-	-
214.	*Tupaia glis*	KTR089	Oil palm plantation	Johor, Malaysia	Male	Adult	-	-	-
215.	*Tupaia glis*	KTR090	Oil palm plantation	Johor, Malaysia	Male	Adult	-	-	-

216.	*Tupaia glis*	KTR091	Oil palm plantation	Johor, Malaysia	Male	Adult	*Borrelia* sp. (RF)	UM-SNI14	OP375128	
217.	*Tupaia glis*	KTR100	Oil palm plantation	Johor, Malaysia	Male	Adult	-	-	-	
			**Total number of individuals**					47		217
